# Association of four non-insulin-based insulin resistance surrogate markers with colorectal cancer risk: a large-scale prospective cohort study using the UK Biobank

**DOI:** 10.3389/fendo.2026.1848724

**Published:** 2026-07-10

**Authors:** Ye Ding, Jie Wang, Nan Wang, Jinyu Zhu, Xiaoxiao Wang, Peibei Duan

**Affiliations:** 1 School of Nursing, Nanjing University of Chinese Medicine, Nanjing, China; 2 Department of Nursing, Jiangsu Province Hospital of Chinese Medicine, Affiliated Hospital of Nanjing University of Chinese Medicine, Nanjing, China; 3 Department of Endocrinology, Jiangsu Province Hospital of Chinese Medicine, Affiliated Hospital of Nanjing University of Chinese Medicine, Nanjing, China; 4 Clinical Research Institute, Affiliated Hospital of Nanjing University of Chinese Medicine, Nanjing, China

**Keywords:** colorectal cancer, decision curve analysis, insulin resistance, METS-IR, risk prediction, surrogate markers, TyG-BMI

## Abstract

**Background:**

Colorectal cancer (CRC) remains a persistent global health challenge, with a rising incidence strongly linked to metabolic dysfunction. Insulin resistance (IR), a hallmark of metabolic dysregulation, is a well-established driver of tumorigenesis and cancer progression. However, the relative predictive value of distinct non-insulin-based surrogate markers of IR for CRC risk assessment remains poorly defined.

**Methods:**

This prospective study analyzed data from 385,206 participants in the UK Biobank. Non-insulin-based surrogate markers evaluated included the TyG index, TyG-BMI, the TG/HDL-C ratio, and METS-IR. Cox proportional hazards models estimated hazard ratios (HRs) and 95% confidence intervals (CIs) for incident CRC. Restricted cubic splines (RCS) assessed dose-response relationships. Incremental predictive value and clinical utility were evaluated via C-index increments and decision curve analysis (DCA). Robustness was verified through extensive subgroup and sensitivity analyses.

**Results:**

Over a median follow-up of 13.8 years, 5,175 incident CRC cases were documented. All four IR surrogates were independently and positively associated with CRC risk (all *P* < 0.001). In the fully adjusted model, comparing extreme quartiles (Q4 vs. Q1), the HRs (95% CIs) were 1.16 (1.07-1.26) for the TyG index, 1.20 (1.11-1.31) for TyG-BMI, 1.09 (1.01-1.19) for the TG/HDL-C ratio, and 1.21 (1.11-1.32) for METS-IR. RCS revealed significant non-linear relationships for the TyG index and METS-IR, whereas linear trends were observed for the remaining markers in the fully adjusted model. Notably, adding METS-IR and TyG-BMI to the base model yielded the most significant improvements in discrimination (both C-index increments = 0.0015; *P* < 0.001). Furthermore, DCA confirmed sustained net clinical benefits across practical thresholds. Subgroup analyses revealed significant interactions between the IR surrogate markers and age, sex, and smoking status (*P*-interaction ≤ 0.05). Multiple sensitivity analyses, including excluding cases diagnosed within the first two years, confirmed the findings’ robustness.

**Conclusions:**

Elevated non-insulin-based IR surrogates are robust predictors of CRC risk. The BMI-integrated indices, METS-IR and TyG-BMI, demonstrated superior risk stratification performance. These low-cost, readily available tools may facilitate the early identification of high-risk individuals and inform evidence-based primary prevention strategies.

## Introduction

Colorectal cancer (CRC) persists as a major global public health challenge, contributing substantially to the worldwide cancer burden. According to the latest GLOBOCAN 2022 estimates, CRC is now the third most commonly diagnosed malignancy and the second leading cause of cancer-related mortality worldwide (1). In 2022 alone, there were an estimated 1.93 million new CRC cases and 904,000 associated deaths globally. Projections indicate that the global CRC burden will rise by 60% by 2030, with annual incident cases exceeding 2.2 million and related deaths surpassing 1.1 million (1). This escalating burden is driven primarily by population aging and the increasing adoption of Western-style lifestyle patterns in low- and middle-income countries (2). Beyond genetic predisposition, dietary factors, sedentary behavior, and metabolic dysregulation are well-established, key modifiable drivers of the rising incidence of this malignancy (3).

Metabolic syndrome (MetS) is a complex metabolic disorder characterized by central obesity, hypertension, hyperglycemia, and dyslipidemia (4). Previous meta-analyses indicate that individuals with MetS face a 25% to 34% higher risk of developing CRC compared to those without this condition (5). Among the multiple components of MetS, insulin resistance (IR) is widely recognized as a key pathophysiological factor associated with metabolic dysfunction contributing to CRC development (6, 7).

Pathophysiologically, IR is characterized by impaired biological responses to insulin in peripheral tissues, leading to compensatory hyperinsulinemia. Chronic elevation of circulating insulin levels promotes tumor cell proliferation and suppresses apoptosis by activating the insulin/insulin-like growth factor-1 (IGF-1) axis and inducing chronic low-grade inflammation (8, 9). Although the hyperinsulinemic-euglycemic clamp remains the “gold standard” for quantifying IR (10), its high cost, invasive nature, and complex procedure severely limit its clinical applicability (11). Similarly, the commonly used Homeostatic Model Assessment of Insulin Resistance (HOMA-IR) requires measuring fasting insulin levels, which is costly to assess routinely and has limited diagnostic value for individuals receiving insulin therapy or those with beta-cell dysfunction. Consequently, this test is rarely included in routine large-scale population screening programs (12, 13). To overcome these obstacles, researchers have developed and validated several non-insulin-based surrogate markers. These include the triglyceride-glucose (TyG) index (14), the TyG-body mass index (TyG-BMI) (15), the triglyceride-to-high-density lipoprotein cholesterol (TG/HDL-C) ratio (16), and the metabolic score for insulin resistance (METS-IR) (17). Calculated from readily accessible anthropometric and biochemical parameters, these indices have demonstrated high sensitivity and specificity in predicting metabolic diseases and various malignancies, including breast and esophageal cancers (18, 19).

Although recent studies have preliminarily explored the association between IR surrogate markers and CRC risk, existing evidence is largely confined to single markers or specific populations. Crucially, there is a notable lack of direct head-to-head comparisons regarding the predictive efficacy of these distinct markers for incident CRC (20, 21). This limitation hinders the identification of the most robust metabolic warning markers, thereby restricting the optimization of early risk stratification strategies. To address this critical knowledge gap, we leveraged the UK Biobank’s large-scale prospective cohort data to systematically conduct the first longitudinal comparison of four mainstream IR markers — TyG index, TyG-BMI, the TG/HDL-C ratio, and METS-IR—within a unified analytical framework. By utilizing a massive sample size and comprehensive follow-up, this study aims to evaluate the independent associations of these markers with CRC risk and precisely identify whether integrated indices (combining both anthropometric and biochemical parameters) offer superior predictive utility. Ultimately, this multidimensional comparative analysis seeks to provide robust evidence for selecting the most clinically valuable metabolic prediction tools, significantly enhancing the accuracy of early CRC risk stratification and informing targeted primary prevention strategies.

## Materials and methods

### Data source and study population

This study utilized data from the UK Biobank, a large-scale prospective cohort that recruited over 500,000 participants aged 37 to 73 years across the United Kingdom between 2006 and 2010. The data used in this study were directly obtained from the official UK Biobank Resource under Application Number 419342. Access was granted following the approval of a formal research proposal, and the study was conducted in strict accordance with the UK Biobank’s terms of use and the Material Transfer Agreement. The original project received ethical approval from the North West Multi-Centre Research Ethics Committee (MREC), and all participants provided written informed consent. All data provided to researchers were de-identified and anonymized to protect participant privacy. The current analysis utilized the August 2025 data release from the UK Biobank, ensuring the inclusion of the most recent clinical and biochemical updates available for the analytical cohort.

**Figure 1:**
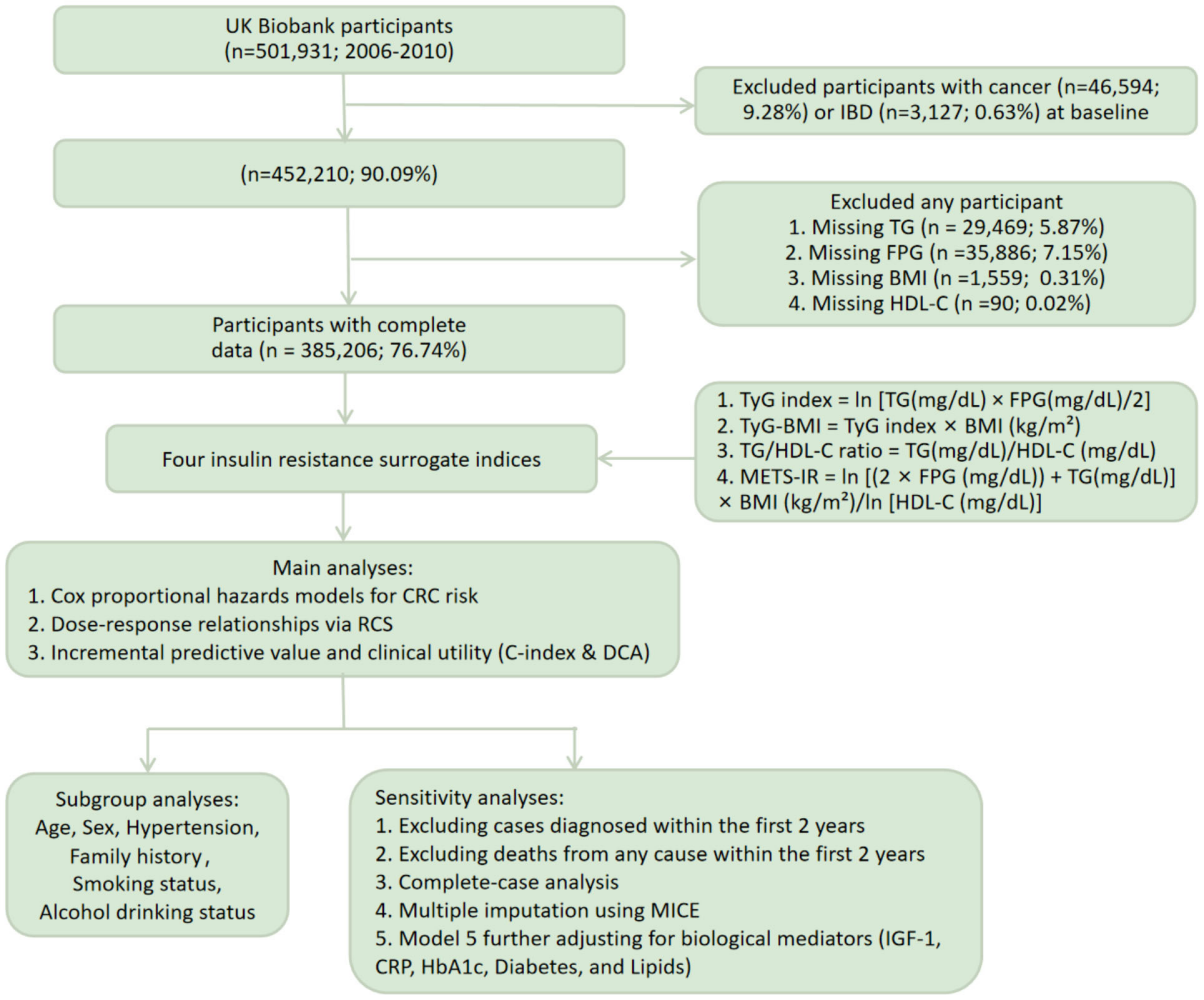
Flowchart of participant selection and study design.

From the original cohort, we defined the study population using strict exclusion criteria (1): participants with a history of any malignant tumor (n = 46,594) or inflammatory bowel disease (IBD) (n = 3,127) prior to the baseline assessment; and (2) participants with missing baseline data for key variables, including triglycerides (*n* = 29,469), fasting plasma glucose (FPG; *n* = 35,886), body mass index (BMI; *n* = 1,559), or high-density lipoprotein cholesterol (HDL-C; *n* = 90). The final analytical cohort comprised 385,206 participants. The detailed flowchart of participant selection is illustrated in **Figure 1**.

### Definitions of four insulin resistance surrogate indices

All participants underwent a standardized baseline assessment (2006–2010), during which peripheral venous blood samples were collected. The exact duration of fasting prior to phlebotomy was systematically recorded. All blood specimens were processed within 24 hours of collection and analyzed at a central laboratory accredited by the UK National External Quality Assessment Service (UK NEQAS), using a Beckman Coulter AU5800 automated clinical chemistry analyzer. The primary biochemical analytes included serum triglycerides (TG), glucose (representing fasting plasma glucose [FPG] levels), and HDL-C, all measured in serum aliquots using standardized protocols.

During the same baseline assessment, trained research staff performed standardized measurements of standing height (to the nearest 0.1 cm) and body weight (to the nearest 0.1 kg) using calibrated medical-grade equipment, in accordance with UK Biobank standard operating procedures (SOPs). Body mass index (BMI) was calculated as weight in kilograms divided by height in meters squared (kg/m²). To assess non-insulin-based insulin resistance (IR), we calculated four surrogate indices using the following established formulas: TyG index = ln [TG (mg/dL) × FPG (mg/dL)/2]; TyG-BMI = TyG index × BMI (kg/m²); TG/HDL-C ratio = TG (mg/dL)/HDL-C (mg/dL); METS-IR = ln [(2 × FPG (mg/dL)) + TG (mg/dL)] × BMI (kg/m²)/ln [HDL-C (mg/dL)].

### Ascertainment of outcome

The primary outcome of this study was incident CRC. Incident cases were identified through linkage to national cancer registries and death registries. supplemented by participant self-reported medical history. CRC cases were defined according to the International Classification of Diseases, Tenth Revision (ICD-10) codes C18–C20. The follow-up period for each participant commenced on the date of baseline assessment and ended at the earliest occurrence of the following events: (1) first diagnosis of CRC; (2) death from any cause; or (3) the administrative censoring date (December 31, 2022).

### Selection of covariates

Baseline covariates for this study were obtained through standardized touchscreen questionnaires, nurse-led interviews, physical examinations, and biological sampling. Sociodemographic factors included age, sex, ethnicity, education level, annual household income, employment status, and the Townsend Deprivation Index (TDI). Lifestyle-related variables encompassed smoking status, alcohol drinking status, physical activity (categorized using the International Physical Activity Questionnaire [IPAQ]), sleep duration, dietary habits (intake of cereal, processed meat, fruit, and vegetables) and the use of vitamin D supplements. Clinical, medication, and screening history included a family history of CRC, hypertension, and diabetes, the regular use of lipid-lowering medications and non-steroidal anti-inflammatory drugs (NSAIDs) and bowel cancer screening history (encompassing fecal occult blood testing, sigmoidoscopy, and colonoscopy). Relevant laboratory and clinical parameters encompassed fasting duration, glycated hemoglobin (HbA1c), total cholesterol (TC), low-density lipoprotein cholesterol (LDL-C), insulin-like growth factor-1 (IGF-1), and C-reactive protein (CRP), the latter of which served as a clinical indicator of systemic low-grade inflammation.

### Statistical analyses

Due to the lack of consensus on clinical cutoff values and potential population heterogeneity, we divided the four IR surrogate markers into quartiles (Q1–Q4), with the lowest quartile (Q1) serving as the reference. Baseline characteristics were summarized across these quartiles. Continuous variables were expressed as mean ± standard deviation (SD) or median (interquartile range [IQR]) based on their distribution (assessed via the Kolmogorov-Smirnov test), while categorical variables were presented as frequencies (percentages). Differences across groups were assessed using one-way analysis of variance (ANOVA) or the Kruskal-Wallis test for continuous variables, and Pearson’s Chi-square test for categorical variables. Kaplan-Meier curves were constructed to estimate the cumulative incidence of CRC across quartiles, with differences evaluated using the log-rank test.

To quantify the independent associations between IR surrogate markers and CRC risk, Cox proportional hazards models were used to estimate hazard ratios (HRs) and 95% confidence intervals (CIs). Four progressively adjusted models were constructed: Model 1 was unadjusted; Model 2 adjusted for sociodemographic factors (age, sex, ethnicity, education level, annual household income, employment status, and the Townsend Deprivation Index); Model 3 further incorporated lifestyle and dietary factors (smoking and drinking status, physical activity level, sleep duration, and dietary intakes of cereal, fruit, vegetables, and processed meat) as well as vitamin D supplementation. Finally, Model 4 further incorporated clinical, medication, and screening history, including hypertension, family history of CRC, the regular use of lipid-lowering medications and NSAIDs, bowel cancer screening history (including fecal occult blood testing, sigmoidoscopy, and colonoscopy), and fasting duration. The selection of covariates and the stepwise adjustment strategy were strictly informed by a Directed Acyclic Graph (DAG) (**Supplementary Figure S1**). The DAG identified sociodemographic and lifestyle factors as ancestral confounders, while clinical and biochemical indicators—including diabetes history, HbA1c, TC, LDL-C, IGF-1, and CRP—were categorized as potential downstream mediators. Accordingly, our primary multivariable model (Model 4) focused on estimating the total metabolic effect by adjusting solely for confounders and clinical history. We further constructed Model 5 as a sensitivity analysis, incorporating the full set of potential mediators to evaluate the independent direct effect of IR surrogate markers beyond established metabolic and inflammatory pathways. Linear trends (*P* for trend) were assessed by treating the median of each quartile as a continuous variable. Furthermore, HRs corresponding to a one-standard-deviation (1-SD) increase in each IR metric were calculated. To facilitate risk stratification in clinical practice, we determined the optimal cutoff point for each IR indicator using the maximally selected rank statistics method (via the *surv_cutpoint* function in the R package *survminer*). Participants were categorized into high-level (> cutpoint) and low-level (≤ cutpoint, reference) groups and analyzed using the same four Cox models.

To further investigate potential dose-response relationships, we employed restricted cubic splines (RCS) integrated into the Cox regression, with three knots placed at the 10th, 50th, and 90th percentiles of the exposure distribution. When a significant non-linear association was detected (*P* for non-linearity < 0.05), a two-stage segmented linear regression model was applied. The optimal breakpoint—representing the critical threshold where the slope of the CRC risk trajectory significantly changes—was automatically estimated using the Newton-Raphson algorithm via the *segmented* package in R.

To evaluate the incremental value of incorporating IR markers into established risk assessment systems, we utilized Harrell’s C-index and the likelihood ratio test (LRT). A base clinical model was constructed comprising all covariates from Model 4. The four IR markers were individually added to this base model. The improvement in discriminatory ability was quantified by the change in the C-index (ΔC-index), with the LRT assessing the statistical significance of overall model fit improvement. Finally, decision curve analysis (DCA) was conducted at the cohort level to assess the practical net clinical benefit of the updated models across various threshold probabilities, compared against the default strategies of “treat all” and “treat none”.

As for data integrity, covariates with a missingness rate of < 5% were imputed using the median for continuous variables and the mode for categorical variables. For covariates with missingness rates ≥ 5%, a separate “missing” category was created to preserve the sample size and account for potential systematic differences.

### Subgroup and sensitivity analyses

Finally, to validate the robustness of the primary findings and explore potential population heterogeneity, we conducted extensive subgroup analyses and interaction tests. These analyses were stratified by age (< 50 years vs. ≥ 50 years), sex (male vs. female), hypertension status (yes vs. no), smoking and alcohol drinking status (never, previous, or current), and family history of CRC (yes vs. no). Multiplicative interaction terms were introduced into the fully adjusted Cox models to assess the statistical significance of effect modifications across strata.

Furthermore, we performed five robust sensitivity analyses to ensure the stability of our findings. First, to minimize potential reverse causality, we conducted a landmark analysis by excluding participants who developed CRC within the first two years of follow-up. Second, to account for the potential confounding effects of early-onset ill health, individuals who died from any cause within the first two years of follow-up were also excluded. Third, a complete-case analysis was performed by excluding individuals with any missing covariate data to evaluate the impact of imputation techniques on the final estimates. Fourth, to address potential selection bias and maintain statistical power, we applied multiple imputation by chained equations (MICE) to handle missing covariates, comparing these results with our primary analysis for consistency. Fifth, to evaluate the independent direct effect of IR surrogate markers and explore potential mediating pathways, we constructed an additional sensitivity model (Model 5). This model further adjusted for clinical and biochemical mediators identified by our Directed Acyclic Graph (DAG), including diabetes history, glycated hemoglobin (HbA1c), total cholesterol (TC), low-density lipoprotein cholesterol (LDL-C), insulin-like growth factor-1 (IGF-1), and C-reactive protein (CRP).

All statistical analyses were performed using R software (version 4.5.1; R Foundation for Statistical Computing). A two-sided *P* < 0.05 was considered statistically significant.

## Results

### Baseline characteristics

Between 2006 and 2010, the UK Biobank recruited 501,931 participants. After applying the predefined exclusion criteria, the final analytical cohort comprised 385,206 participants (**Figure 1**; **Supplementary Table S1**). During a median follow-up of 13.8 years (interquartile range [IQR]: 13.1–14.5), yielding approximately 5.18 million person-years, 5,175 incident CRC cases were documented. The overall crude incidence rate of CRC was 99.9 per 100,000 person-years.

**Table 1** summarizes the baseline characteristics of the study population stratified by TyG index quartiles. Compared with the lowest quartile (Q1), participants in higher TyG quartiles were more likely to be male, to be current or previous smokers, and to have a higher prevalence of hypertension and diabetes (*P* < 0.001). Additionally, these groups exhibited higher levels of BMI, fasting plasma glucose, glycated hemoglobin, total cholesterol, triglycerides, and low-density lipoprotein cholesterol, along with lower HDL-C concentrations (*P* < 0.001). Detailed baseline characteristics stratified by TyG-BMI, the TG/HDL-C ratio, and METS-IR quartiles are presented in the Supplementary Material (**Supplementary Tables S2**–**S4**).

**Table 1:** Baseline characteristics of the study population according to quartiles of the TyG index.

Characteristic	Total	Quartiles of TyG index				P value
		Q1 ≤8.31	Q2 (8.31–8.68]	Q3 (8.68–9.08]	Q4 >9.08	
Number of participants	385206	96303	96300	96301	96302	
Age, years	56.24 ± 8.09	54.03 ± 8.27	56.48 ± 8.03	57.25 ± 7.85	57.18 ± 7.80	<0.001
Male, n(%)	181710 (47.2%)	32424 (33.7%)	40921 (42.5%)	48995 (50.9%)	59370 (61.6%)	<0.001
TDI	-1.31 ± 3.06	-1.33 ± 3.07	-1.40 ± 3.01	-1.36 ± 3.04	-1.15 ± 3.12	<0.001
Ethnicity, n(%)						<0.001
White	363548 (94.4%)	89588 (93.0%)	91224 (94.7%)	91560 (95.1%)	91176 (94.7%)	
Others	21658 (5.6%)	6715 (7.0%)	5076 (5.3%)	4741 (4.9%)	5126 (5.3%)	
Qualification, n(%)						<0.001
College	125874 (32.7%)	37184 (38.6%)	32391 (33.6%)	29380 (30.5%)	26919 (28.0%)	
Non-College	259332 (67.3%)	59119 (61.4%)	63909 (66.4%)	66921 (69.5%)	69383 (72.0%)	
Employment, n(%)						<0.001
Yes	228329 (59.3%)	65173 (67.7%)	56927 (59.1%)	53719 (55.8%)	52510 (54.5%)	
No	156877 (40.7%)	31130 (32.3%)	39373 (40.9%)	42582 (44.2%)	43792 (45.5%)	
Income, n(%)						<0.001
Less than 18,000	72918 (18.9%)	14549 (15.1%)	17634 (18.3%)	19332 (20.1%)	21403 (22.2%)	
18,000 to 30,999	82769 (21.5%)	18879 (19.6%)	20692 (21.5%)	21693 (22.5%)	21505 (22.3%)	
31,000 to 51,999	87008 (22.6%)	23031 (23.9%)	22004 (22.8%)	21201 (22.0%)	20772 (21.6%)	
52,000 to 100,000	68910 (17.9%)	20579 (21.4%)	17342 (18.0%)	15900 (16.5%)	15089 (15.7%)	
Greater than 100,000	18413 (4.8%)	6375 (6.6%)	4750 (4.9%)	3884 (4.0%)	3404 (3.5%)	
Missing	55188 (14.3%)	12890 (13.4%)	13878 (14.4%)	14291 (14.8%)	14129 (14.7%)	
Smoking status, n(%)						<0.001
Never	213314 (55.4%)	58428 (60.7%)	54954 (57.1%)	52042 (54.0%)	47890 (49.7%)	
Previous	131141 (34.0%)	29556 (30.7%)	31837 (33.1%)	33836 (35.1%)	35912 (37.3%)	
Current	40751 (10.6%)	8319 (8.6%)	9509 (9.9%)	10423 (10.8%)	12500 (13.0%)	
Drinking status, n(%)						<0.001
Never	16877 (4.4%)	3854 (4.0%)	4090 (4.2%)	4236 (4.4%)	4697 (4.9%)	
Previous	13515 (3.5%)	3031 (3.1%)	3190 (3.3%)	3382 (3.5%)	3912 (4.1%)	
Current	354814 (92.1%)	89418 (92.9%)	89020 (92.4%)	88683 (92.1%)	87693 (91.1%)	
Sleep Duration, hours/day	7.15 ± 1.10	7.12 ± 1.02	7.14 ± 1.07	7.16 ± 1.11	7.17 ± 1.18	<0.001
IPAQ, n(%)						<0.001
Low	54942 (14.3%)	11408 (11.8%)	12657 (13.1%)	14169 (14.7%)	16708 (17.3%)	
Moderate	121166 (31.5%)	30311 (31.5%)	30489 (31.7%)	30274 (31.4%)	30092 (31.2%)	
High	122632 (31.8%)	34763 (36.1%)	31535 (32.7%)	29554 (30.7%)	26780 (27.8%)	
Missing	86466 (22.4%)	19821 (20.6%)	21619 (22.4%)	22304 (23.2%)	22722 (23.6%)	
Processed meat intake, n(%)						<0.001
Never	35358 (9.2%)	11740 (12.2%)	9365 (9.7%)	7786 (8.1%)	6467 (6.7%)	
Less than once a week	117287 (30.4%)	32820 (34.1%)	31211 (32.4%)	28507 (29.6%)	24749 (25.7%)	
Once a week	112572 (29.0%)	27408 (28.3%)	28028 (28.9%)	28620 (29.5%)	28516 (29.4%)	
2–4 times a week	105079 (27.3%)	21435 (22.3%)	24385 (25.3%)	27610 (28.7%)	31649 (32.9%)	
5–6 times a week	12448 (3.2%)	2471 (2.6%)	2770 (2.9%)	3149 (3.3%)	4058 (4.2%)	
Once or more daily	3218 (0.8%)	622 (0.6%)	716 (0.7%)	815 (0.8%)	1065 (1.1%)	
Fruit intake (pieces/day)	2[1, 3]	2[1, 3]	2[1, 3]	2[1, 3]	2[1, 3]	<0.001
Cereal intake (bowls/week)	4.49 ± 2.80	4.45 ± 2.84	4.57 ± 2.77	4.55 ± 2.77	4.40 ± 2.81	<0.001
Vegetable intake (tablespoons/day)	2 [2, 3]	2 [2, 3]	2 [2, 3]	2 [2, 3]	2 [2, 3]	0.001
Vitamin D intake, n (%)	14771 (3.8%)	4181 (4.3%)	3910 (4.1%)	3516 (3.7%)	3164 (3.3%)	<0.001
Diabetes, n(%)	19881 (5.2%)	1872 (1.9%)	2587 (2.7%)	4285 (4.4%)	11137 (11.6%)	<0.001
Hypertension, n(%)	109749 (28.5%)	17854 (18.5%)	24746 (25.7%)	30272 (31.4%)	36877 (38.3%)	<0.001
Family history of CRC, n(%)						<0.001
No	343793 (89.2%)	86702 (90.0%)	85970 (89.3%)	85740 (89.0%)	85381 (88.7%)	
Yes	41413 (10.8%)	9601 (10.0%)	10330 (10.7%)	10561 (11.0%)	10921 (11.3%)	
BMI, kg/m^2^	27.42 ± 4.61	25.20 ± 3.94	26.85 ± 4.35	28.13 ± 4.49	29.50 ± 4.52	<0.001
NSAIDs, n(%)	103854 (27.0%)	23518 (24.4%)	24823 (25.8%)	26233 (27.2%)	29280 (30.4%)	<0.001
Lipid-lowering drug, n(%)	66186 (17.2%)	10116 (10.5%)	13959 (14.5%)	17706 (18.4%)	24405 (25.3%)	<0.001
Cancer screening, n(%)	113352 (29.4%)	23769 (24.7%)	29031 (30.1%)	30294 (31.5%)	30258 (31.4%)	<0.001
HbA1c, mmol/mol	35.20 [32.90, 37.70]	34.30 [31.90, 36.20]	35.10 [32.70, 37.10]	35.30 [33.20, 37.90]	36.30 [34.10,39.80]	<0.001
Glucose, mmol/L	4.93[4.60, 5.31]	4.73[4.43, 5.04]	4.89[4.59, 5.21]	4.97[4.65, 5.33]	5.18[4.79, 5.83]	<0.001
TG, mmol/L	1.48[1.04, 2.14]	0.84[0.71, 0.96]	1.26[1.13, 1.39]	1.78[1.59, 1.99]	2.80[2.38, 3.48]	<0.001
TC, mmol/L	5.69 ± 1.11	5.35 ± 0.98	5.64 ± 1.05	5.81 ± 1.11	5.96 ± 1.22	<0.001
HDL-C, mmol/L	1.44 ± 0.37	1.67 ± 0.38	1.52 ± 0.35	1.38 ± 0.31	1.21 ± 0.27	<0.001
LDL-C, mmol/L	3.56 ± 0.85	3.22 ± 0.72	3.53 ± 0.79	3.71 ± 0.85	3.77 ± 0.91	<0.001
CRP, mg/L	1.32 [0.66,2.68]	0.88 [0.44,1.77]	1.25 [0.62,2.46]	1.46 [0.78,2.97]	1.78 [0.98,3.45]	<0.001
IGF-1, nmol/L	21.40 ± 5.41	21.77 ± 5.42	21.56 ± 5.35	21.39 ± 5.39	20.88 ± 5.42	<0.001
Fasting time, hours	3[2, 4]	3[2, 5]	3[3, 5]	3[3, 4]	3[2, 4]	<0.001

BMI, body mass index; HbA1c, glycated hemoglobin; TG, triglycerides; TC, total cholesterol; HDL-C, high-density lipoprotein cholesterol; LDL-C, low-density lipoprotein cholesterol; TyG, triglyceride-glucose index; CRP, C-reactive protein; IGF-1, insulin-like growth factor-1.

### Association between IR surrogates and CRC risk

**Figure 2:**
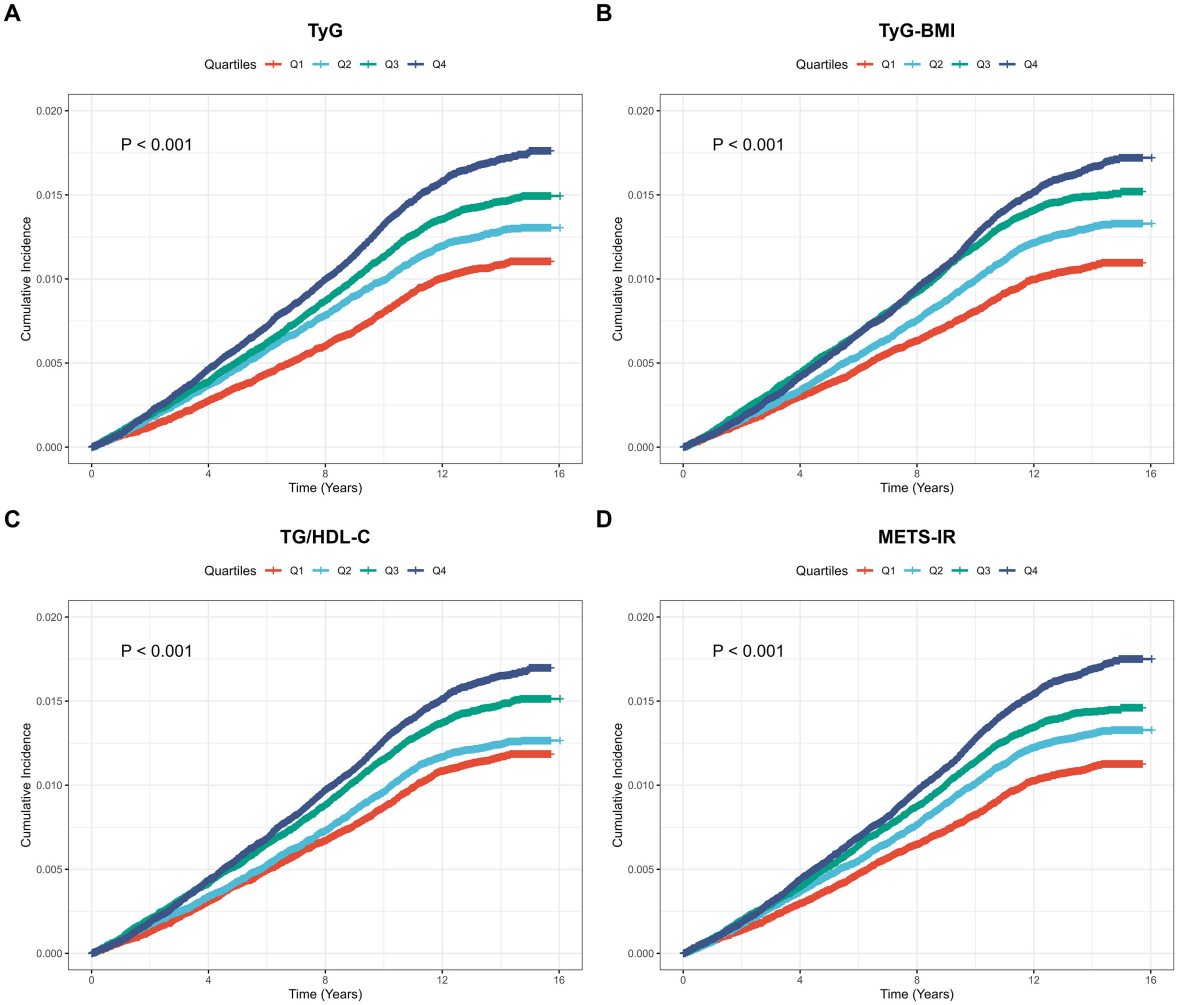
Kaplan-Meier curves for the cumulative incidence of CRC according to quartiles of the TyG index **(A)**, TyG-BMI **(B)**, the TG/HDL-C ratio **(C)**, and METS-IR **(D)**.

First, we estimated the cumulative incidence of CRC across groups stratified by quartiles of the four IR surrogate markers using Kaplan-Meier (KM) analysis. As shown in **Figure 2**, participants with elevated levels of these IR-related indicators exhibited significantly higher CRC incidence rates (all log-rank test *P* < 0.001).

Next, to further quantify these associations, we constructed progressively adjusted Cox proportional hazards models (**Table 2**). When analyzed as continuous variables, all four IR surrogate markers—TyG index, TyG-BMI, the TG/HDL-C ratio, and METS-IR—demonstrated a significant positive association with CRC risk. In the unadjusted analysis (Model 1), elevated levels of these markers were strongly associated with increased CRC risk (*P* < 0.001). These associations remained robust after adjusting for demographic and lifestyle factors (Models 2 and 3). After full adjustment for socioeconomic status, lifestyle factors, medical history, medication use, and screening history (Model 4), the hazard ratios (HRs) and 95% confidence intervals (CIs) per 1-standard-deviation (1-SD) increase were: 1.07 (1.04–1.10) for TyG index, 1.09 (1.06–1.12) for TyG-BMI, 1.06 (1.03–1.09) for the TG/HDL-C ratio, and 1.09 (1.06–1.13) for METS-IR.

**Table 2:** Associations of the four IR surrogate markers with the risk of incident CRC across progressively adjusted models.

Type	Model 1	Model 2	Model 3	Model 4
	HR (95% CI)	HR (95% CI)	HR (95% CI)	HR (95% CI)
TyG				
Q1	Ref	Ref	Ref	Ref
Q2	1.19 (1.09-1.29)^c^	0.98 (0.90-1.07)	0.98 (0.90-1.06)	0.98 (0.90-1.06)
Q3	1.35 (1.25-1.47)^c^	1.04 (0.96-1.13)	1.03 (0.95-1.12)	1.03 (0.95-1.12)
Q4	1.58 (1.46-1.71)^c^	1.19 (1.10-1.29)^c^	1.16 (1.07-1.26)^c^	1.16 (1.07-1.26)^c^
P for trend	<0.001	<0.001	<0.001	<0.001
Per SD increase	1.19 (1.16-1.22)^c^	1.08 (1.05-1.11)^c^	1.07 (1.04-1.10)^c^	1.07 (1.04–1.10)^c^
KM cutpoint				
≤8.68	Ref	Ref	Ref	Ref
>8.68	1.34 (1.27-1.42)^c^	1.13 (1.07-1.19)^c^	1.11 (1.05-1.17)^c^	1.11 (1.05-1.17)^c^
TyG-BMI				
Q1	Ref	Ref	Ref	Ref
Q2	1.22 (1.12-1.32)^c^	1.01 (0.93-1.10)	1.00 (0.92-1.08)	0.99 (0.91-1.08)
Q3	1.40 (1.29-1.51)^c^	1.10 (1.01-1.19)^a^	1.07 (0.98-1.16)	1.07 (0.98-1.16)
Q4	1.55 (1.43-1.68)^c^	1.26 (1.16-1.37)^c^	1.21 (1.11-1.31)^c^	1.20 (1.11-1.31)^c^
P for trend	<0.001	<0.001	<0.001	<0.001
Per SD increase	1.16 (1.13-1.19)^c^	1.11 (1.08-1.14)^c^	1.09 (1.06-1.12)^c^	1.09 (1.06–1.12)^c^
KM cutpoint				
≤218.40	Ref	Ref	Ref	Ref
>218.40	1.40 (1.32-1.49)^c^	1.19 (1.12-1.26)^c^	1.15 (1.09-1.23)^c^	1.15 (1.08–1.22)^c^
TG/HDL				
Q1	Ref	Ref	Ref	Ref
Q2	1.07 (0.99-1.16)	0.93 (0.85-1.01)	0.93 (0.85-1.01)	0.93 (0.85-1.01)
Q3	1.28 (1.18-1.38)^c^	1.02 (0.94-1.11)	1.01 (0.93-1.10)	1.01 (0.93-1.10)
Q4	1.42 (1.31-1.53)^c^	1.12 (1.03-1.21)^b^	1.09 (1.01-1.19)^a^	1.09 (1.01-1.19)^a^
P for trend	<0.001	<0.001	0.003	0.004
Per SD increase	1.13 (1.10-1.16)^c^	1.07 (1.04-1.10)^c^	1.06 (1.03-1.09)^c^	1.06 (1.03-1.09)^c^
KM cutpoint				
≤2.61	Ref	Ref	Ref	Ref
>2.61	1.32 (1.25-1.40)^c^	1.14 (1.08-1.21)^c^	1.12 (1.06-1.19)^c^	1.12 (1.06–1.19)^c^
METS-IR				
Q1	Ref	Ref	Ref	Ref
Q2	1.18 (1.09-1.29)^c^	1.01 (0.93-1.10)	1.00 (0.92-1.09)	1.00 (0.92-1.09)
Q3	1.31 (1.20-1.42)^c^	1.05 (0.97-1.14)	1.03 (0.95-1.12)	1.03 (0.94-1.12)
Q4	1.53 (1.42-1.66)^c^	1.26 (1.16-1.37)^c^	1.22 (1.12-1.32)^c^	1.21 (1.11-1.32)^c^
P for trend	<0.001	<0.001	<0.001	<0.001
Per SD increase	1.16 (1.13-1.19)^c^	1.11 (1.08-1.14)^c^	1.10 (1.06-1.13)^c^	1.09 (1.06–1.13)^c^
KM cutpoint				
≤44.79	Ref	Ref	Ref	Ref
>44.79	1.35 (1.28-1.43)^c^	1.26 (1.19-1.33)^c^	1.23 (1.16-1.30)^c^	1.23 (1.15-1.30)^c^

CRC, colorectal cancer; SD, standard deviation; HR, hazard ratio; 95% CI, 95% confidence interval;.

Model 1: unadjusted.

Model 2: adjusted for age, sex, annual household income, ethnicity, education level, employment status, and TDI

Model 3: Further adjusted for physical activity level, sleep duration, smoking status, drinking status, and dietary intakes of fruit, cereal, vegetables, and processed meat and vitamin D supplementation.

Model 4: Further adjusted for hypertension, family history of CRC, fasting duration, the regular use of lipid-lowering medications and NSAIDs, and bowel cancer screening history.

a: P < 0.05, b: P < 0.01, c: P < 0.001.

To further elucidate these associations, subjects were categorized into quartiles (Q1–Q4) based on the baseline level of each indicator. Compared with the lowest quartile (Q1, reference), participants in the highest quartile (Q4) demonstrated significantly elevated CRC risk across all models. In the fully adjusted analysis (Model 4), the HRs (95% CIs) for Q4 relative to Q1 were: 1.16 (1.07-1.26) for TyG index, 1.20 (1.11-1.31) for TyG-BMI, 1.09 (1.01-1.19) for the TG/HDL-C ratio, and 1.21 (1.11-1.32) for METS-IR. Notably, all indicators demonstrated significant linear trends across quartiles (*P* for trend < 0.001 for the TyG, TyG-BMI, and METS-IR; *P* for trend = 0.004 for the TG/HDL-C ratio).

Finally, recognizing the need for practical risk stratification in clinical settings, we dichotomized participants based on the optimal survival cutpoints determined by the maximally selected rank statistics method. The optimal thresholds were identified as 8.68 for TyG index, 218.40 for TyG-BMI, 2.61 for the TG/HDL-C ratio, and 44.79 for METS-IR. Participants with levels exceeding these thresholds (the high-level group) faced a significantly higher risk of incident CRC compared to those below the thresholds (the low-level reference group). In the fully adjusted Model 4, the corresponding HRs (95% CIs) were 1.11 (1.05-1.17, *P* < 0.001) for TyG index, 1.15 (1.08-1.22, *P* < 0.001) for TyG-BMI, 1.12 (1.06-1.19, *P* < 0.001) for the TG/HDL-C ratio, and 1.23 (1.15-1.30, *P* < 0.001) for METS-IR. Consistent with the continuous and quartile analyses, the BMI-integrated indices (METS-IR and TyG-BMI) continued to exhibit the most pronounced risk-discriminating capacity.

### RCS analysis investigating the association between IR surrogates and CRC risk

**Figure 3:**
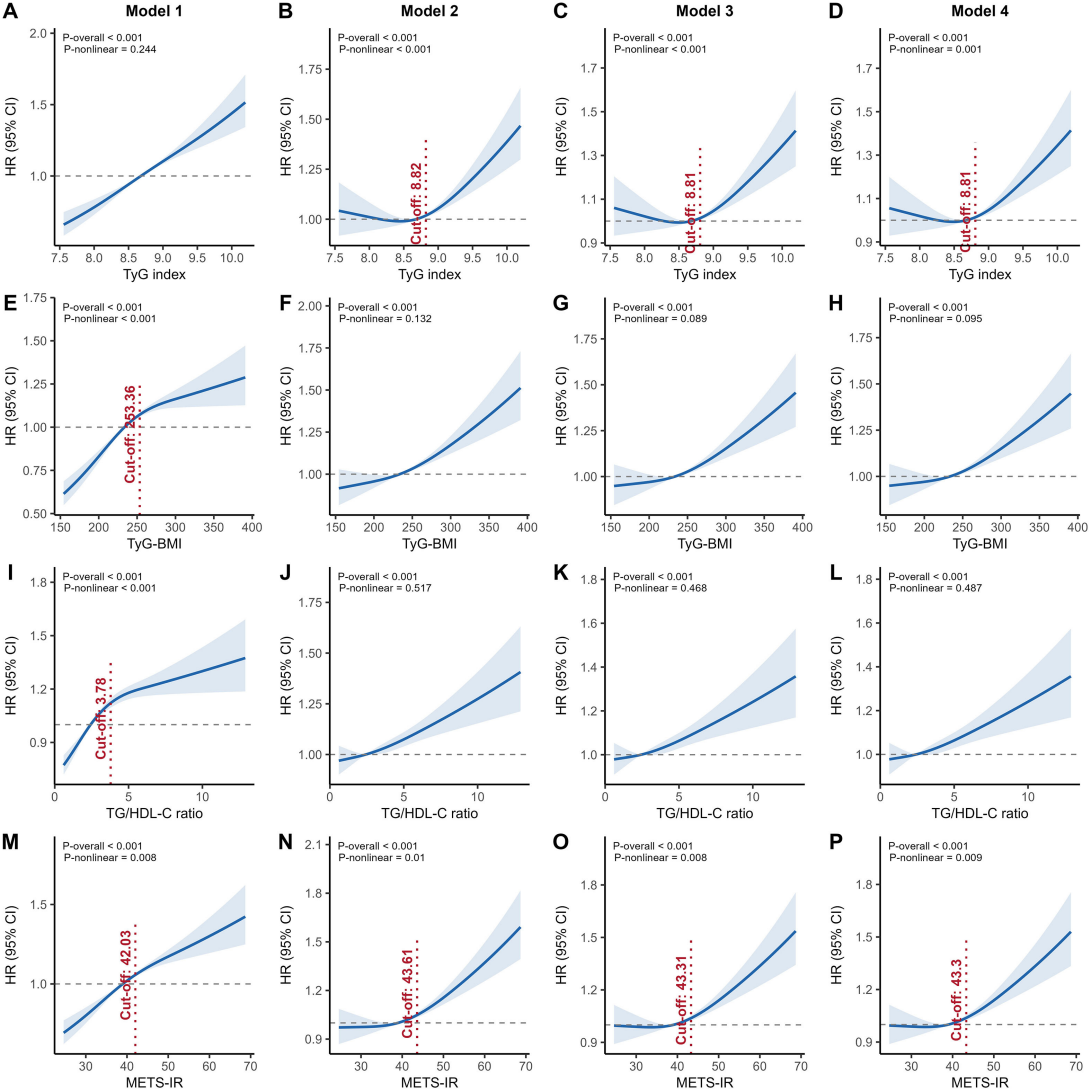
Dose-response relationships between the four IR surrogate markers and incident CRC risk. Subplots represent the **(A–D)** TyG index, **(E–H)** TyG-BMI index, **(I–L)** TG/HDL ratio, and **(M–P)** METS-IR. Within each row, columns from left to right correspond to Model 1, Model 2, Model 3, and Model 4, respectively. Solid lines indicate hazard ratios (HRs) estimated by restricted cubic splines (RCS) with three knots, and shaded areas represent 95% confidence intervals. Model 1 was unadjusted. Model 2 was adjusted for age, sex, ethnicity, education level, annual household income, employment status, and the Townsend Deprivation Index. Model 3 was further adjusted for lifestyle and dietary factors (smoking and drinking status, physical activity level, sleep duration, and dietary intakes of cereal, fruit, vegetables, and processed meat) and vitamin D supplementation. Model 4 (the fully adjusted model) further incorporated clinical, medication, and screening history, including hypertension, family history of CRC, the regular use of lipid-lowering medications and NSAIDs, bowel cancer screening history (including fecal occult blood testing, sigmoidoscopy, and colonoscopy), and fasting duration.

To further investigate the dose-response relationships between the four IR surrogate markers—the TyG index, TyG-BMI, the TG/HDL-C ratio, and METS-IR—and the risk of incident CRC, we employed multivariable-adjusted restricted cubic spline (RCS) models across progressively adjusted Models 1 to 4 (**Figures 3A–P**). As shown in **Figure 3**, while the associations generally evolved as covariates were incrementally added (**Figures 3A–C, E–G, I–K, M–O**), all four metabolic indices in the fully adjusted Model 4 exhibited significant overall positive associations with CRC risk (all *P* for overall < 0.001). However, their dose-response trajectories diverged considerably.

Notably, both the TyG index and METS-IR maintained significant non-linear relationships with incident CRC risk in the fully adjusted Model 4 (*P* for non-linearity = 0.001 and 0.009, respectively). The RCS curves for these two indices exhibited typical “J”-shaped trajectories, indicating pronounced threshold effects (**Figures 3D, P**). Subsequent two-stage segmented linear regression identified critical risk inflection points at 8.81 for the TyG index and 43.3 for METS-IR. Below these thresholds, the risk increase was relatively gradual, whereas beyond them, the risk escalated sharply. In contrast, TyG-BMI and the TG/HDL-C ratio demonstrated primarily linear dose-response relationships (**Figures 3H, L**). For these two indices, tests for non-linearity in Model 4 were not statistically significant (TyG-BMI: P = 0.095; the TG/HDL-C ratio: P = 0.487).

Therefore, among the studied markers, the TyG index and METS-IR retained independent non-linear threshold characteristics in the fully adjusted model. Conversely, TyG-BMI and the TG/HDL-C ratio acted primarily as continuous, linear risk factors, whereby any incremental increase in these indices corresponded to a proportional increase in CRC risk.

### Incremental predictive value and clinical utility of IR surrogate indices

We evaluated the incremental predictive value of the four IR surrogate indices when added to a comprehensive baseline clinical model (Harrell’s C-index = 0.6788). The addition of any single IR index significantly improved the global model fit (Likelihood Ratio Test [LRT], all P < 0.001). As shown in **Table 3**, METS-IR and TyG-BMI provided the highest incremental value, both increasing the C-index to 0.6803 (both ΔC-index = 0.0015). The TyG index and the TG/HDL-C ratio also yielded modest but statistically significant improvements in model discrimination, with ΔC-indices of 0.0010 and 0.0009, respectively.

**Table 3:** Incremental predictive value of incorporating IR surrogate indices into the baseline clinical model for incident CRC.

Added_Biomarker	Base C-Index	New C-Index	Delta C-Index	LRT P-Value
TyG	0.6788	0.6798	0.0010	<0.001
TyG-BMI	0.6788	0.6803	0.0015	<0.001
TG/HDL-C	0.6788	0.6797	0.0009	<0.001
METS-IR	0.6788	0.6803	0.0015	<0.001

Note: The base model includes age, sex, ethnicity, education level, annual household income, employment status, Townsend Deprivation Index, smoking and drinking status, physical activity level, sleep duration, dietary intakes (processed meat, fruit, cereal and vegetables), vitamin D supplementation, hypertension, family history of CRC, fasting duration, the regular use of lipid-lowering medications and NSAIDs, and bowel cancer screening history.

**Figure 4:**
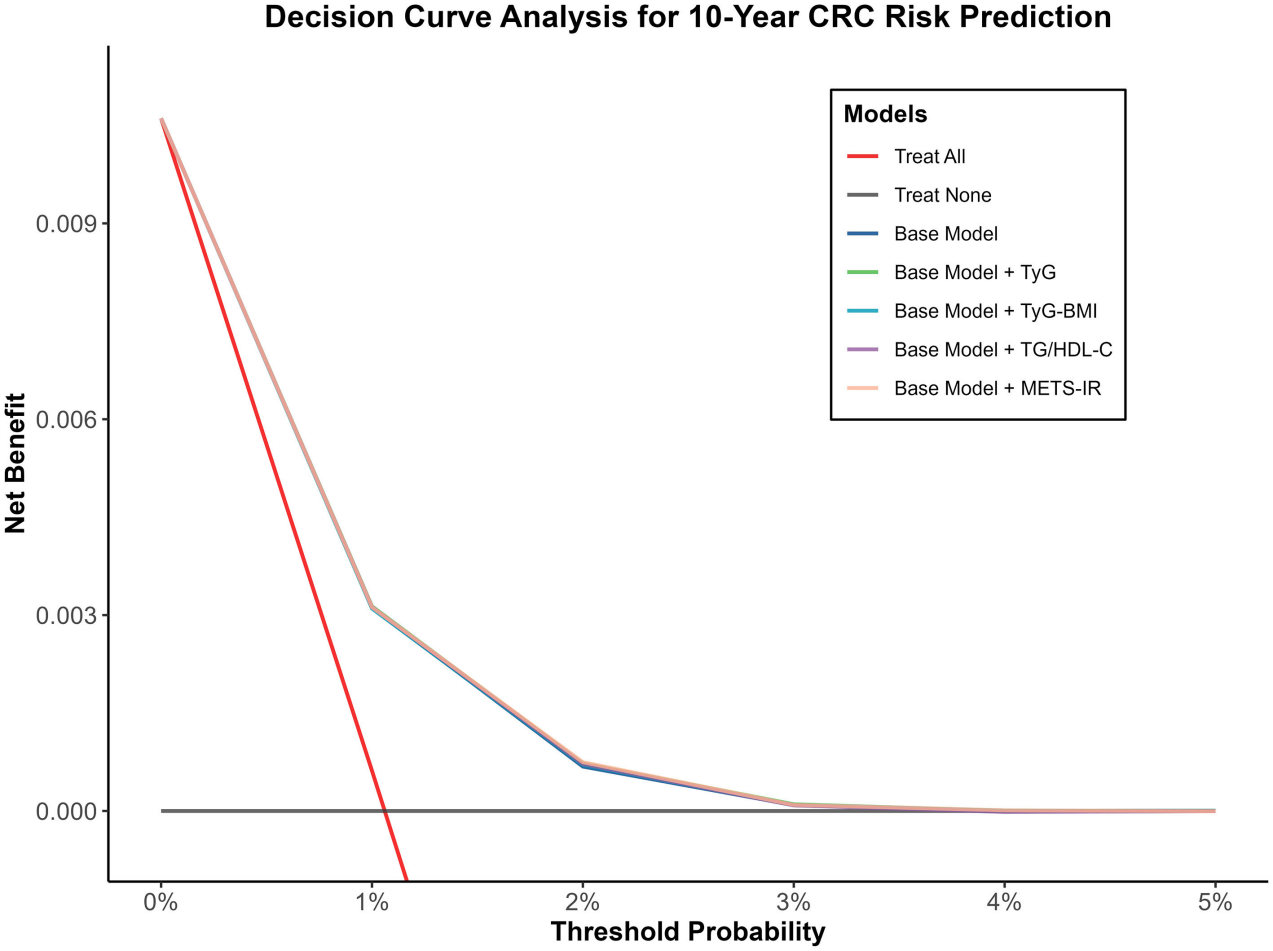
Decision curve analysis (DCA) for predicting the 10-year risk of incident CRC.

To translate these statistical improvements into practical clinical applications, we performed a Decision Curve Analysis (DCA) for the 10-year CRC risk (**Figure 4**). Across a clinically relevant range of threshold probabilities (approximately 0.5% to 3.5%), all models—including the baseline model and the IR-enhanced models—consistently provided higher net benefits compared to the extreme “universal treatment” or “no treatment” strategies. Although the net benefit curves visually overlap due to the robust performance of the baseline model and the low absolute incidence of CRC, the sustained net benefit within the practical threshold range confirms that integrating the IR biomarker effectively preserves and optimizes the clinical utility of the existing risk assessment framework.

### Subgroup and sensitivity analyses

**Figure 5:**
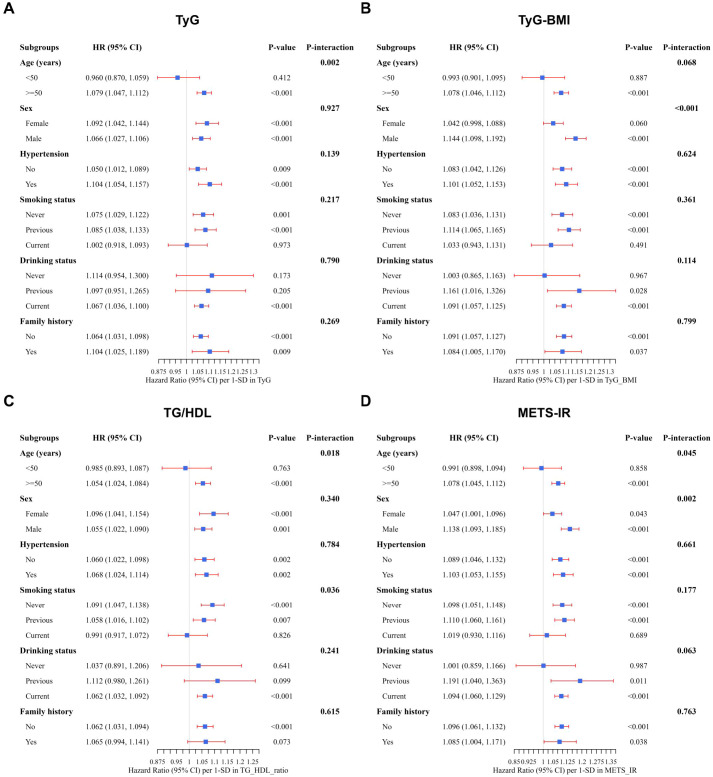
Subgroup analyses of the associations between the four IR surrogate markers—**(A)** the TyG index, **(B)** TyG-BMI, **(C)** the TG/HDL ratio, and **(D)** METS-IR—and the risk of incident CRC. Hazard ratios (HRs) and 95% CIs were calculated using the fully adjusted Model 4. Interactions were tested for age, sex, hypertension, smoking status, drinking status, and family history of CRC.

To explore potential population heterogeneity and the stability of our findings, we conducted extensive subgroup analyses stratified by age, sex, hypertension, smoking status, drinking status, and family history of CRC. As shown in **Figure 5**, while the positive associations between the four IR surrogates and incident CRC were generally consistent across several strata, significant multiplicative interactions were identified for age, sex, and smoking status (**Figures 5A–D**). Regarding age, significant interactions were observed for the TyG index (*P* for interaction = 0.002), the TG/HDL-C ratio (*P* for interaction = 0.018), and METS-IR (*P* for interaction = 0.045). Robust positive associations were documented only in the older subgroup (≥ 50 years), whereas these associations were not statistically significant in the younger subgroup (< 50 years), despite the documentation of 449 incident cases in this stratum. Similarly, significant interactions were observed between sex and the BMI-integrated indices: TyG-BMI (*P* for interaction < 0.001) and METS-IR (*P* for interaction = 0.002), with stronger associations observed in males (TyG-BMI: HR = 1.144, 95% CI: 1.098–1.192; METS-IR: HR = 1.138, 95% CI: 1.093–1.185). Furthermore, lifestyle factors also exhibited specific interaction effects: a significant interaction was detected between smoking status and the TG/HDL-C ratio (*P* for interaction = 0.036), showing a stronger association among never-smokers (HR = 1.091; 95% CI: 1.047–1.138). No other significant interactions were detected (all *P* for interaction > 0.05).

Beyond patient-level subgroups, we conducted sub-site analyses to determine whether the associations varied between colon cancer (C18, n = 3,536) and rectal cancer (C19–C20, n = 1,950), acknowledging that 311 patients were diagnosed with both. In the fully adjusted Model 4, all four IR surrogate markers exhibited robust positive associations with colon cancer risk, with significantly elevated risks in the highest quartiles (e.g., METS-IR HR (Q4 vs. Q1): 1.29, 95% CI: 1.16–1.43; TyG-BMI HR (Q4 vs. Q1): 1.25, 95% CI: 1.12–1.38; all *P* for trend < 0.001; **Supplementary Table S5**). Conversely, the associations were notably attenuated for rectal cancer. When categorized into quartiles, none of the IR markers reached statistical significance for the highest group (Q4) in the rectal cancer stratum, and the trend tests were generally non-significant (all *P* for trend > 0.05). However, when treated as continuous variables, per 1-SD increases in the BMI-integrated indices—TyG-BMI (HR: 1.06, 95% CI: 1.01–1.11, P = 0.017) and METS-IR (HR: 1.05, 95% CI: 1.00–1.11, P = 0.038)—remained significantly associated with increased rectal cancer risk. Overall, these findings indicate that the oncogenic influence of metabolic dysfunction is substantially more pronounced in the colon than in the rectum.

The robustness of our primary findings was further confirmed through five rigorous sensitivity analyses, all of which yielded results highly consistent with the main analysis (**Supplementary Tables S6**–**S10**). Specifically, the positive associations between the four non-insulin-based IR surrogate markers and incident CRC risk remained stable and statistically significant after: (1) excluding participants who developed CRC within the first two years of follow-up (**Supplementary Table S6**); (2) excluding those who died from any cause within the same period (**Supplementary Table S7**); (3) performing a complete-case analysis (**Supplementary Table S8**); (4) utilizing multiple imputation via MICE to address missing covariates (**Supplementary Table S9**); and (5) constructing an additional sensitivity model (Model 5) that further adjusted for a comprehensive suite of potential mediators, including diabetes, HbA1c, total cholesterol, LDL-C, IGF-1, and CRP. Notably, this DAG-informed model confirmed that the studied markers provide significant independent predictive value beyond established metabolic and inflammatory pathways (**Supplementary Table S10**). Collectively, these results demonstrate that the identified metabolic-cancer associations are resilient to potential biases from reverse causality, data missingness, or the mediating effects of downstream clinical markers.

## Discussion

In this large-scale prospective study using data from the UK Biobank, we established that four non-insulin-based surrogate markers of insulin resistance (IR)—TyG index, TyG-BMI, the TG/HDL-C ratio, and METS-IR—are robustly and independently associated with an increased risk of incident CRC. Beyond identifying these markers as persistent risk factors, our restricted cubic spline (RCS) analysis delineated distinct dose-response trajectories, highlighting a significant nonlinear threshold effect specifically for the TyG index and METS-IR. Furthermore, integrating these IR indices into established clinical models provided statistically significant incremental predictive value and consistent net clinical benefits, as demonstrated by improved C-indices and Decision Curve Analysis (DCA). These associations remained resilient in landmark analyses excluding cases diagnosed within the first two years of follow-up, reinforcing the hypothesis of a genuine temporal sequence between metabolic dysfunction and oncogenesis. Ultimately, our findings suggest that these inexpensive, accessible biomarkers could play a pivotal role in refining CRC risk stratification and promoting proactive metabolic interventions in primary care settings.

Crucially, although all four surrogate markers showed significant positive correlations with CRC risk, their predictive performance exhibited subtle but clinically meaningful differences. Our analysis revealed that indices incorporating anthropometric measures (e.g., METS-IR and TyG-BMI) demonstrated more pronounced associations with CRC risk compared to those based solely on lipid and glycemic parameters (e.g., TyG and the TG/HDL-C ratio). In the fully adjusted model, the hazard ratios (HRs) for the highest quartiles of METS-IR and TyG-BMI were 1.21 and 1.20, respectively, substantially higher than those for TyG (1.16) and the TG/HDL-C ratio (1.09). This discrepancy may stem from the fact that METS-IR and TyG-BMI simultaneously integrate body mass index (BMI) and biochemical markers, thereby comprehensively reflecting both systemic insulin resistance and excessive visceral fat accumulation. Adipose tissue functions not only as an energy storage depot but also as an active endocrine organ, secreting pro-inflammatory cytokines and adipokines that actively promote the formation of a pro-tumorigenic colorectal microenvironment (22–24). Consistent with these findings, our evaluation of clinical incremental value demonstrated highly significant improvements in global model fit upon adding these IR indices (LRT *P* < 0.001), with METS-IR providing the highest incremental utility. We acknowledge that the absolute increments in the C-index (ranging from 0.001 to 0.0015) were modest, and the DCA curves visually tracked the baseline model closely. However, this phenomenon—often referred to as the “AUC paradox”—is expected in large-scale, population-based predictive epidemiology (25). Our baseline clinical model was already highly optimized with over 20 potent risk factors (base C-index = 0.6788); in such comprehensive models, it is mathematically formidable for any single novel biomarker to drive a massive leap in absolute discrimination (26). Furthermore, CRC is a profoundly complex multifactorial disease, and IR represents only one component of the carcinogenic puzzle. Thus, the modest absolute ΔC-index does not diminish the epidemiological significance of these markers. Instead, the consistent LRT results and the sustained net clinical benefits observed in the DCA across practical screening thresholds—a method increasingly endorsed by top-tier medical literature to evaluate true clinical utility beyond pure statistical discrimination (27)—confirm that indices like METS-IR capture unique, independent pathophysiological variance. Given that these surrogate markers do not require fasting insulin testing, they offer an economical, efficient, and clinically accessible strategy to refine current risk assessment frameworks and identify high-risk populations for CRC, particularly in resource-limited settings. Furthermore, we addressed potential over-adjustment bias using a Directed Acyclic Graph (DAG) approach. Since factors like diabetes and HbA1c can act as mediators, our primary Model 4 estimated the total metabolic effect by excluding these variables. Notably, our sensitivity analysis (Model 5), which further incorporated these potential mediators into the model, yielded hazard ratios remarkably consistent with our primary findings. This remarkable consistency suggests that these IR surrogates capture a fundamental, intrinsic oncogenic risk that operates independently of overt clinical metabolic diseases. It further reinforces the potential of these markers for early risk stratification, as their predictive value remains robust across varying stages of metabolic deterioration.

The significant interaction between age and IR surrogate markers observed in our study provides a nuanced understanding of colorectal tumorigenesis. While these markers robustly predict CRC risk in individuals aged 50 and older, their predictive value appeared to be attenuated in the younger subgroup (< 50 years). This discrepancy may be partly attributed to the distinct etiologies of early-onset (EO-CRC) versus late-onset CRC (LO-CRC). EO-CRC is frequently characterized by a stronger genetic predisposition and unique molecular features—such as higher rates of microsatellite instability or specific epigenetic signatures—which may develop independently of traditional metabolic-driven pathways (28, 29). In contrast, LO-CRC often reflects the long-term cumulative impact of insulin resistance, compensatory hyperinsulinemia, and systemic chronic inflammation—pathophysiological processes that are potentially captured more effectively by the surrogate markers analyzed in this study (30). Furthermore, the lack of significant association in the younger cohort may also be influenced by the ‘healthy volunteer’ bias inherent in the UK Biobank, where younger participants often exhibit fewer cumulative metabolic insults compared to the general population (31). Nonetheless, our findings underscore that monitoring metabolic health is particularly critical for CRC risk stratification as individuals transition into older age, providing a window for primary prevention before the peak incidence of LO-CRC (1). Furthermore, our sub-site analysis revealed that while metabolic dysregulation is a broad risk factor, its impact appears more localized to the colon. The observed site-specific nuances align with previous evidence suggesting that the colon may be more physiologically sensitive to hyperinsulinemia and circulating IGF-1 levels than the rectum (32). These differences may be attributed to divergent embryological origins, variations in fecal transit time and bile acid exposure, and the distinct microbiota composition between the two sites (33). However, the attenuated associations observed in the rectal cancer subgroup should be interpreted with caution. Given the substantially lower number of incident cases in the rectal cancer stratum (n = 1,950) compared to the colon cancer stratum (n = 3,536), we cannot exclude the possibility that the lack of significance in categorical analyses was partly due to limited statistical power to detect more modest effect sizes.

Another compelling finding of this study is the significant sexual dimorphism observed in the association between IR surrogate markers—particularly the BMI-integrated indices (TyG-BMI and METS-IR)—and incident CRC risk. Our interaction analysis revealed a notable sex-specific divergence, where the predictive value of these markers was markedly stronger in male participants compared to females (interaction P < 0.05). Given that direct measurements of circulating sex hormones were not available in the UK Biobank biochemical assessment, our mechanistic interpretations remain hypothesis-generating and should be viewed as speculative. However, several established biological pathways provide a plausible context for this observation. First, the sexually dimorphic distribution of adipose tissue could potentially play a role. Males exhibit a strong predilection for accumulating visceral adipose tissue (VAT), whereas females predominantly store fat in subcutaneous compartments. Visceral fat is significantly more metabolically active, inherently more lipolytic, and serves as a major driver of systemic insulin resistance and chronic low-grade inflammation—both of which directly promote colorectal tumorigenesis (34–36). By incorporating BMI as a proxy for adiposity, METS-IR and TyG-BMI may capture the deleterious effects of central obesity, which are often more pronounced in men. Second, the potential protective endocrine role of estrogen warrants consideration based on external evidence. Epidemiological and experimental studies have suggested that estrogen may inhibit colorectal tumorigenesis through multiple synergistic mechanisms, including the modulation of bile acid metabolism and maintenance of intestinal barrier integrity via estrogen receptor-beta (ERβ) signaling (37, 38). Although we could not empirically verify these hormonal interactions within our cohort, it is possible that the lack of such estrogenic protection, combined with a higher baseline prevalence of metabolic dysfunction, contributes to the heightened vulnerability observed in male participants. Collectively, these anatomical and hormonal hypotheses provide a biological framework for the observed sex-specific risk estimates, though further studies incorporating sex-hormone profiling are required to substantiate these links.

Notably, we observed that for several IR surrogates, the increased risk of incident CRC was most pronounced in the highest quartile (Q4). This risk distribution pattern suggests a potential threshold effect, where the pro-tumorigenic impact of chronic metabolic dysfunction becomes clinically manifest primarily at higher levels of insulin resistance. Such a finding is consistent with our restricted cubic spline (RCS) analysis (**Figure 3**), which demonstrated significant non-linear relationships for both the TyG index and METS-IR, characterized by sharp risk escalations beyond their respective inflection points (8.81 for the TyG index and 43.3 for METS-IR). Although hazard ratios for the middle quartiles (Q2 and Q3) did not always reach statistical significance when compared to Q1, the highly significant linear trend tests across all markers (*P* for trend < 0.001) confirm a robust overall monotonic relationship. From a clinical perspective, this ‘Q4-driven significance’ indicates that identifying individuals in the top 25% of the IR surrogate distribution may offer the highest yield for targeted colorectal screening and proactive primary prevention strategies (39).

Beyond the aforementioned sex-specific factors, the fundamental biological mechanisms linking insulin resistance (IR)—as reflected by these non-insulin-based surrogate markers—to the development of CRC are inherently multifaceted. This oncogenic process is potentially characterized by complex, synergistic interactions among compensatory hyperinsulinemia, the insulin/insulin-like growth factor-1 (IGF-1) axis, and chronic low-grade inflammation. A central pathophysiological mechanism likely involves the insulin/IGF system. Chronic hyperinsulinemia, the defining hallmark of severe IR, directly suppresses the hepatic synthesis of insulin-like growth factor-binding proteins (particularly IGFBP-1 and IGFBP-2), thereby significantly increasing the bioavailability of circulating free IGF-1 (8, 40). Crucially, as IGF-1 and specific lipid fractions function as pivotal downstream mediators or manifestations within this causal pathway, we justified our covariate selection using a Directed Acyclic Graph (DAG) and performed sensitivity analyses excluding these mediators to avoid over-adjustment bias. The remarkable consistency of the results confirms that these IR surrogates capture a fundamental oncogenic risk that precedes overt clinical metabolic deterioration. Both insulin and unbound IGF-1 potently activate the phosphoinositide 3-kinase (PI3K)/Akt and mitogen-activated protein kinase (MAPK) signaling cascades by binding to their respective homologous receptors on the surface of colorectal epithelial cells. The aberrant activation of these pathways not only intensely stimulates cellular proliferation but also critically suppresses apoptosis, thereby facilitating tumorigenesis and early malignant progression (41, 42). Furthermore, the profound lipid metabolism abnormalities inherently captured by these surrogate markers—such as elevated circulating triglycerides and depressed HDL cholesterol (HDL-C) levels—are intimately linked to systemic lipotoxicity and chronic inflammation. This metabolic dysregulation triggers the excessive secretion of pro-inflammatory cytokines (e.g., tumor necrosis factor-alpha [TNF-α] and interleukin-6 [IL-6]) from dysfunctional adipose tissue. These circulating cytokines induce profound oxidative stress and consequent DNA damage in the intestinal epithelium, thereby establishing and perpetuating a highly permissible microenvironment for colorectal tumor initiation and clonal expansion (43, 44). In summary, elevated TyG, TyG-BMI, the TG/HDL-C ratio, and METS-IR indices do not merely reflect an individual’s deteriorating metabolic state; rather, they serve as integrated, measurable proxies for a profoundly complex, pro-carcinogenic endocrine and inflammatory milieu.

Extrapolating from these shared systemic mechanisms, the predictive utility of non-insulin-based IR surrogate markers likely extends beyond CRC to a broader spectrum of malignancies. Emerging evidence has linked elevated TyG index and METS-IR indices to an increased risk of other gastrointestinal cancers, including esophageal, gastric and pancreatic cancers (18, 45, 46). The chronic inflammatory state and hyperinsulinemia reflected by these markers are thought to establish a similarly permissible environment in these tissues. Furthermore, these IR surrogates have demonstrated significant predictive potential for breast cancer, particularly in postmenopausal women, where the interplay between insulin signaling and estrogen pathways accelerates mammary tumorigenesis (19). These emerging data suggest that TyG-related and BMI-integrated indices may serve as broad-spectrum metabolic warning markers for various early-onset GI and obesity-related cancers, reflecting the systemic nature of metabolic-driven oncogenesis (47). Future pan-cancer longitudinal studies are warranted to fully delineate the multi-organ oncogenic potential of these IR surrogates.

Our findings are highly consistent with existing evidence while significantly expanding the application of non-insulin-based IR surrogate markers in predicting CRC risk. Consistent with recent prospective analyses across diverse populations, such as the Japanese cohort study by Okamura et al. (21) and the large-scale Korean studies by Son et al. (48) and Kityo et al. (49) — this study confirms that the TyG index is a reliable predictor of incident CRC. A recent systematic review and meta-analysis further supports these findings, highlighting the robust positive correlation between elevated TyG levels and increased CRC susceptibility globally (50). However, the critical novelty of our study lies in providing a comprehensive head-to-head comparison of multiple indicators within a single, unified analytical framework. Unlike previous studies focusing solely on the TyG index or the TG/HDL-C ratio (e.g., Liu et al. (51)), our data from the UK Biobank provide robust evidence that assessment systems integrating anthropometric measures—particularly METS-IR and TyG-BMI—yield superior predictive value. This finding is particularly crucial when exploring the “obesity paradox” and the impact of differential fat distribution on metabolic health. Although some historical data (e.g., the Me-Can project (52)) suggested that BMI-adjusted indicators might exhibit weakened associations with obesity-related cancers, both our current study and recent prospective data from Jiang et al. (20) compellingly indicate that these associations are robust and independent of baseline BMI. Notably, METS-IR exhibited the highest hazard ratio (HR) and incremental predictive utility in our fully adjusted models. Furthermore, unlike retrospective or case-control designs relying on propensity score matching (e.g., Hu et al. (53)), the rigorous prospective design of the UK Biobank cohort, coupled with our landmark sensitivity analyses excluding cases diagnosed within the first two years of follow-up, ensures a clear temporal sequence between metabolic exposure and oncological outcome. By demonstrating that baseline IR surrogate markers robustly predict long-term CRC incidence risk, our findings underscore the clinical promise of these biomarkers as pragmatic, early, non-invasive screening tools. Despite differences in racial backgrounds and methodologies among existing landmark studies, this consistent body of evidence indicates that insulin resistance represents a universal and profoundly modifiable risk factor for CRC. Consequently, TyG-related markers—and particularly BMI-integrated indices like METS-IR—serve as highly practical, cost-effective tools for early risk stratification and targeted intervention in primary care settings.

This study possesses several notable strengths. First, to our knowledge, it represents the most comprehensive prospective investigation to date comparing multiple non-insulin-based surrogate markers of insulin resistance (IR) with incident CRC risk within a single, massive cohort. The UK Biobank provided an unprecedentedly large sample size and long-term follow-up, ensuring robust statistical power to detect subtle associations and conduct extensive subgroup analyses (54). Second, the prospective design, combined with landmark analyses excluding cases diagnosed within the first two years of follow-up, significantly mitigated the risk of reverse causality (protopathic bias), confirming that metabolic dysregulation temporally precedes clinical malignancy (55). Third, a major strength lies in our multi-dimensional methodological rigor. By moving beyond traditional Cox proportional hazards models to incorporate RCS for non-linear threshold identification and DCA for clinical utility evaluation, we provide a holistic assessment of both statistical discrimination and practical application—a depth of analysis largely absent in previous literature. Finally, the “insulin-free” nature of these biomarkers represents a key clinical innovation. Unlike the gold-standard hyperinsulinemic-euglycemic clamp or HOMA-IR, which require expensive and specialized fasting insulin assays, these markers rely solely on routine, universally available glucose, lipid, and anthropometric panels. This democratization of IR assessment offers a highly cost-effective and scalable strategy for global primary CRC prevention, particularly in resource-limited environments. Nevertheless, several limitations must be acknowledged. First, the IR surrogate measures relied on biochemical and anthropometric data from a single baseline time point, which cannot capture dynamic longitudinal trajectories or reflect long-term metabolic fluctuations over the 13.8-year median follow-up period (56). Second, despite our rigorous and extensive adjustment for sociodemographic, lifestyle, and clinical variables, residual confounding from unmeasured or imprecisely measured factors—such as specific dietary components, environmental exposures, or gut microbiota composition—cannot be entirely ruled out in any observational study (57). Third, reliance on self-reported data for certain lifestyle covariates (e.g., physical activity, dietary habits) may introduce inherent recall or measurement biases (58). Fourth, the predominantly European ancestry (>94%) and well-documented “healthy volunteer” bias of the UK Biobank limits the generalizability of our findings to other populations, such as Asian or Middle Eastern groups. Given that metabolic architectures vary significantly across ethnicities—for instance, the “thin-fat” phenotype in Asians characterized by high IR at lower BMI levels—the optimal risk thresholds for markers like TyG or METS-IR may shift in different ethnic contexts (59). Furthermore, the UK Biobank lacked baseline data on fasting insulin or OGTT, precluding direct comparison with HOMA-IR. However, this limitation actually highlights the clinical utility of our findings; since insulin assays are often cost-prohibitive or unavailable in large-scale screenings, the evaluated “insulin-free” surrogates offer a pragmatic and scalable alternative for global CRC risk stratification.

## Conclusion

In summary, this large-scale prospective study of 385,206 UK Biobank participants indicates that non-insulin-based surrogate markers of insulin resistance (IR)—namely the TyG index, TyG-BMI, the TG/HDL-C ratio, and METS-IR—are independently and positively associated with the risk of incident CRC. Notably, composite indices integrating anthropometric measures of obesity—specifically the Metabolic Score for Insulin Resistance (METS-IR) and the triglyceride-glucose index combined with BMI (TyG-BMI)—demonstrated superior predictive value, particularly among male participants. These findings underscore a robust association between metabolic dysfunction and colorectal tumorigenesis, indicating that these cost-effective, readily accessible surrogate markers may serve as powerful tools for identifying high-risk individuals in primary care settings.

From a public health perspective, mitigating insulin resistance through targeted lifestyle interventions or pharmacological strategies may represent a highly effective approach for the primary prevention of CRC. Future research should aim to validate these findings across diverse ethnic populations and explore the clinical utility of incorporating these metabolic markers into established CRC screening algorithms.

## Data Availability

The datasets analyzed in this study are available from the UK Biobank resource (www.ukbiobank.ac.uk). Access to these data was granted under Application Number 419342. These data are not publicly available due to participant privacy restrictions and the formal Data Use Agreements stipulated by the UK Biobank, which prohibit the redistribution of individual-level data by researchers. Qualified researchers may apply for access to the same dataset through the UK Biobank Access Management System. Requests to access the datasets should be directed to XW, yfy106@njucm.edu.cn.
