# Supplementary material for: Association of four non-insulin-based insulin resistance surrogate markers with colorectal cancer risk: a large-scale prospective cohort study using the UK Biobank
Source: Front Endocrinol (Lausanne). 2026 Jul 10;17:1848724. doi: 10.3389/fendo.2026.1848724 (PMC13395652; doi:10.3389/fendo.2026.1848724)
Supplement: Supplementary file 1 [file DataSheet1.docx]

[Table S1. ICD-10 codes used to define incident colorectal cancer in the UK Biobank. 2](#_Toc170349306)

[Table S2. Baseline characteristics of the study population according to quartiles of TyG-BMI. 3](#_Toc170349307)

[Table S3. Baseline characteristics of the study population according to quartiles of the TG/HDL-C ratio. 5](#_Toc170349307)

[Table S4. Baseline characteristics of the study population according to quartiles of METS-IR. 7](#_Toc170349307)

[Table S5.Associations between the four IR surrogate markers and incident CRC risk, stratified by anatomical sub-site (colon cancer and rectal cancer), based on the fully adjusted Model 4 9](#_Toc170349307)

[Table S6. Sensitivity analysis for the associations between the four IR surrogate markers and incident CRC risk, excluding participants who developed CRC within the first two years of follow-up. 1](#_Toc170349307)0

[Table S7. Sensitivity analysis for the associations between the four IR surrogate markers and incident CRC risk, excluding participants who died from any cause within the first two years of follow-up. 1](#_Toc170349307)1

[Table S8. Sensitivity analysis for the associations between the four IR surrogate markers and incident CRC risk among participants with complete baseline covariate data (complete-case analysis). 1](#_Toc170349308)2

[Table S9. Sensitivity analysis for the associations between the four IR surrogate markers and incident CRC risk after addressing missing covariates via multiple imputation by chained equations (MICE). 1](#_Toc170349309)3

[Table S10. Sensitivity analysis for the associations between the four IR surrogate markers and incident CRC risk after additional adjustment for potential clinical and biochemical mediators (Model 5). 1](#_Toc170349309)4

[Figure S1. Directed Acyclic Graph (DAG) of the association between insulin resistance (IR) surrogate markers and colorectal cancer (CRC) risk. 1](#_Toc170349309)5

| **Table S1** ICD-10 codes used to define incident colorectal cancer in the UK Biobank | | |
| --- | --- | --- |
| **Cancer Type** | **ICD-10 code** | **Type of colorectal cancer** |
| colorectal cancer | C18 | Malignant neoplasm of colon |
|  | C19 | Malignant neoplasm of rectosigmoid junction |
|  | C20 | Malignant neoplasm of rectum |

| **Table S2** Baseline characteristics of the study population according to quartiles of TyG-BMI. | | | | | | |
| --- | --- | --- | --- | --- | --- | --- |
| **Characteristic** | **Total** | **Quartiles of TyG-BMI** | | | | **P value** |
|  |  | **Q1**  **≤204.80** | **Q2**  **(204.81–233.56]** | **Q3**  **(233.56–267.64]** | **Q4**  **>267.64** |  |
| Number of participants | 385206 | 96302 | 96301 | 96301 | 96302 |  |
| Age, years | 56.24 ± 8.09 | 54.59 ± 8.24 | 56.55 ± 8.08 | 57.03 ± 7.98 | 56.77 ± 7.84 | <0.001 |
| Male, n(%) | 181710 (47.2%) | 30007 (31.2%) | 46264 (48.0%) | 54343 (56.4%) | 51096 (53.1%) | <0.001 |
| TDI | -1.31 ± 3.06 | -1.45 ± 3.00 | -1.54 ± 2.96 | -1.37 ± 3.03 | -0.88 ± 3.21 | <0.001 |
| Ethnicity, n(%) |  |  |  |  |  | <0.001 |
| White | 363548 (94.4%) | 91229 (94.7%) | 90974 (94.5%) | 90494 (94.0%) | 90851 (94.3%) |  |
| Others | 21658 (5.6%) | 5073 (5.3%) | 5327 (5.5%) | 5807 (6.0%) | 5451 (5.7%) |  |
| Qualification, n(%) |  |  |  |  |  | <0.001 |
| College | 125874 (32.7%) | 40030 (41.6%) | 33339 (34.6%) | 28761 (29.9%) | 23744 (24.7%) |  |
| Non-College | 259332 (67.3%) | 56272 (58.4%) | 62962 (65.4%) | 67540 (70.1%) | 72558 (75.3%) |  |
| Employment, n(%) |  |  |  |  |  | <0.001 |
| Yes | 228329 (59.3%) | 62076 (64.5%) | 57059 (59.3%) | 55475 (57.6%) | 53719 (55.8%) |  |
| No | 156877 (40.7%) | 34226 (35.5%) | 39242 (40.7%) | 40826 (42.4%) | 42583 (44.2%) |  |
| Income, n(%) |  |  |  |  |  | <0.001 |
| Less than 18,000 | 72918 (18.9%) | 15001 (15.6%) | 16628 (17.3%) | 18744 (19.5%) | 22545 (23.4%) |  |
| 18,000 to 30,999 | 82769 (21.5%) | 19148 (19.9%) | 21004 (21.8%) | 21378 (22.2%) | 21239 (22.1%) |  |
| 31,000 to 51,999 | 87008 (22.6%) | 22590 (23.5%) | 22260 (23.1%) | 21618 (22.4%) | 20540 (21.3%) |  |
| 52,000 to 100,000 | 68910 (17.9%) | 20024 (20.8%) | 18021 (18.7%) | 16739 (17.4%) | 14126 (14.7%) |  |
| Greater than 100,000 | 18413 (4.8%) | 6323 (6.6%) | 4906 (5.1%) | 4135 (4.3%) | 3049 (3.2%) |  |
| Missing | 55188 (14.3%) | 13216 (13.7%) | 13482 (14.0%) | 13687 (14.2%) | 14803 (15.4% ) |  |
| Smoking status, n(%) |  |  |  |  |  | <0.001 |
| Never | 213314 (55.4%) | 58723 (61.0%) | 54763 (56.9%) | 51134 (53.1%) | 48694 (50.6%) |  |
| Previous | 131141 (34.0%) | 27173 (28.2%) | 31575 (32.8%) | 34924 (36.3%) | 37469 (38.9%) |  |
| Current | 40751 (10.6%) | 10406 (10.8%) | 9963 (10.3%) | 10243 (10.6%) | 10139 (10.5%) |  |
| Drinking status, n(%) |  |  |  |  |  | <0.001 |
| Never | 16877 (4.4%) | 3788 (3.9%) | 3728 (3.9%) | 4138 (4.3%) | 5223 (5.4%) |  |
| Previous | 13515 (3.5%) | 3044 (3.2%) | 2895 (3.0%) | 3162 (3.3%) | 4414 (4.6%) |  |
| Current | 354814 (92.1%) | 89470 (92.9%) | 89678 (93.1%) | 89001 (92.4%) | 86665 (90.0%) |  |
| Sleep Duration, hours/day | 7.15 ± 1.10 | 7.16 ± 1.01 | 7.16 ± 1.04 | 7.15 ± 1.10 | 7.12 ± 1.23 | <0.001 |
| IPAQ, n(%) |  |  |  |  |  | <0.001 |
| Low | 54942 (14.3%) | 10531 (10.9%) | 11948 (12.4%) | 13914 (14.4%) | 18549 (19.3%) |  |
| Moderate | 121166 (31.5%) | 31070 (32.3%) | 30860 (32.0%) | 30502 (31.7%) | 28734 (29.8%) |  |
| High | 122632 (31.8%) | 35383 (36.7%) | 33162 (34.4%) | 30166 (31.3%) | 23921 (24.8%) |  |
| Missing | 86466 (22.4%) | 19318 (20.1%) | 20331 (21.1%) | 21719 (22.6%) | 25098 (26.1%) |  |
| Meat intake (times/week), n(%) |  |  |  |  |  | <0.001 |
| Never | 35358 (9.2%) | 13911 (14.4%) | 8808 (9.1%) | 7005 (7.3%) | 5634 (5.9%) |  |
| Less than once a week | 117287 (30.4%) | 33184 (34.5%) | 30772 (32.0%) | 27942 (29.0%) | 25389 (26.4%) |  |
| Once a week | 111816 (29.0%) | 26012 (27.0%) | 28338 (29.4%) | 28798 (29.9%) | 28668 (29.8%) |  |
| 2-4 times a week | 105079 (27.3%) | 20152 (20.9%) | 24810 (25.8%) | 28416 (29.5%) | 31701 (32.9%) |  |
| 5-6 times a week | 12448 (3.2%) | 2442 (2.5%) | 2862 (3.0%) | 3298 (3.4%) | 3846 (4.0%) |  |
| Once or more daily | 3218 (0.8%) | 601 (0.6%) | 711 (0.7%) | 842 (0.9%) | 1064 (1.1%) |  |
| Fruit intake (pieces/day) | 2[1, 3] | 2[1, 3] | 2[1, 3] | 2[1, 3] | 2[1, 3] | <0.001 |
| Cereal intake (bowls/week) | 4.49 ± 2.8 | 4.63 ± 2.86 | 4.65 ± 2.79 | 4.48 ± 2.76 | 4.21 ± 2.75 | <0.001 |
| Vegetable intake (tablespoons/day) | 2 [2, 3] | 2 [2, 3] | 2 [2, 3] | 2 [2, 3] | 2 [2, 3] | <0.001 |
| Vitamin D intake, n (%) | 14771 (3.8%) | 4810 (5.0%) | 3762 (3.9%) | 3325 (3.5%) | 2874 (3.0%) | <0.001 |
| Diabetes, n(%) | 19881 (5.2%) | 1313 (1.4%) | 2364 (2.5%) | 4255 (4.4%) | 11949 (12.4%) | <0.001 |
| Hypertension, n(%) | 109749 (28.5%) | 13413 (13.9%) | 22238 (23.1%) | 30368 (31.5%) | 43730 (45.4%) | <0.001 |
| Family history of CRC, n(%) |  |  |  |  |  | <0.001 |
| No | 343793 (89.2%) | 86641 (90.0%) | 85942 (89.2%) | 85771 (89.1%) | 85439 (88.7%) |  |
| Yes | 41413 (10.8%) | 9661 (10.0%) | 10359 (10.8%) | 10530 (10.9%) | 10863 (11.3%) |  |
| BMI,kg/m^2^ | 27.42 ± 4.61 | 22.48 ± 1.66 | 25.58 ± 1.39 | 28.21 ± 1.65 | 33.41 ± 3.68 | <0.001 |
| NSAIDs, n(%) | 103854 (27.0%) | 20447 (21.2%) | 23778 (24.7%) | 26829 (27.9%) | 32800 (34.1%) | <0.001 |
| Lipid-lowering drug, n(%) | 66186 (17.2%) | 7374 (7.7%) | 13314 (13.8%) | 18793 (19.5%) | 26705 (27.7%) | <0.001 |
| Cancer screening, n(%) | 113352 (29.4%) | 25374 (26.3%) | 28816 (29.9%) | 29821 (31.0%) | 29341 (30.5%) | <0.001 |
| HbA1c,mmol/mol | 35.20  [32.90, 37.70] | 34.40  [32.10, 36.30] | 35.00  [32.60, 37.00] | 35.20  [33.10, 37.80] | 36.60  [34.30, 40.10] | <0.001 |
| Glucose, mmol/L | 4.93[4.60, 5.31] | 4.80[4.48, 5.11] | 4.90[4.58, 5.23] | 4.96[4.64, 5.33] | 5.09[4.72, 5.65] | <0.001 |
| TG,mmol/L | 1.48 [1.04, 2.14] | 0.96[0.76, 1.25] | 1.35[1.04, 1.78] | 1.75[1.30, 2.35] | 2.22[1.62, 3.07] | <0.001 |
| TC,mmol/L | 5.69 ± 1.11 | 5.55 ± 1.01 | 5.73 ± 1.08 | 5.78 ± 1.14 | 5.68 ± 1.20 | <0.001 |
| HDL-C,mmol/L | 1.44 ± 0.37 | 1.69 ± 0.37 | 1.49 ± 0.34 | 1.36 ± 0.32 | 1.24 ± 0.29 | <0.001 |
| LDL-C,mmol/L | 3.56 ± 0.85 | 3.35 ± 0.76 | 3.60 ± 0.83 | 3.68 ± 0.87 | 3.61 ± 0.90 | <0.001 |
| CRP, mg/L | 1.32 [0.66, 2.68] | 0.70 [0.38, 1.32] | 1.12 [0.61, 2.05] | 1.48 [0.85, 2.76] | 2.43 [1.32, 4.61] | <0.001 |
| IGF-1, nmol/L | 21.40 ± 5.41 | 21.77 ± 5.35 | 22.00 ± 5.27 | 21.69 ± 5.34 | 20.14 ± 5.46 | <0.001 |
| Fasting time, hours | 3[2, 4] | 3[2, 4] | 3[2, 4] | 3[2, 4] | 3[2, 4] | <0.001 |
| **Table S3** Baseline characteristics of the study population according to quartiles of the TG/HDL-C ratio. | | | | | | |
| **Characteristic** | **Total** | **Quartiles of the TG/HDL-C ratio** | | | | **P value** |
|  |  | **Q1**  **≤1.51** | **Q2**  **(1.51–2.43]** | **Q3**  **(2.43–3.97]** | **Q4**  **>3.97** |  |
| Number of participants | 385206 | 96302 | 96301 | 96301 | 96302 |  |
| Age, years | 56.24 ± 8.09 | 55.00 ± 8.18 | 56.40 ± 8.09 | 57.01 ± 7.98 | 56.53 ± 7.99 | <0.001 |
| Male, n(%) | 181710 (47.2%) | 25417 (26.4%) | 38886 (40.4%) | 50778 (52.7%) | 66629 (69.2%) | <0.001 |
| TDI | -1.31 ± 3.06 | -1.43 ± 3.00 | -1.39 ± 3.03 | -1.30 ± 3.06 | -1.12 ± 3.15 | <0.001 |
| Ethnicity, n(%) |  |  |  |  |  | <0.001 |
| White | 363548 (94.4%) | 90567 (94.0%) | 90930 (94.4%) | 91098 (94.6%) | 90953 (94.4%) |  |
| Others | 21658 (5.6%) | 5735 (6.0%) | 5371 (5.6%) | 5203 (5.4%) | 5349 (5.6%) |  |
| Qualification, n(%) |  |  |  |  |  | <0.001 |
| College | 125874 (32.7%) | 37198 (38.6%) | 32055 (33.3%) | 29385 (30.5%) | 27236 (28.3%) |  |
| Non-College | 259332 (67.3%) | 59104 (61.4%) | 64246 (66.7%) | 66916 (69.5%) | 69066 (71.7%) |  |
| Employment, n(%) |  |  |  |  |  | <0.001 |
| Yes | 228329 (59.3%) | 61667 (64.0%) | 56757 (58.9%) | 54550 (56.6%) | 55355 (57.5%) |  |
| No | 156877 (40.7%) | 34635 (36.0%) | 39544 (41.1%) | 41751 (43.4%) | 40947 (42.5%) |  |
| Income, n(%) |  |  |  |  |  | <0.001 |
| Less than 18,000 | 72918 (18.9%) | 14555 (15.1%) | 17935 (18.6%) | 19458 (20.2%) | 20970 (21.8%) |  |
| 18,000 to 30,999 | 82769 (21.5%) | 19191 (19.9%) | 20652 (21.4%) | 21721 (22.6%) | 21205 (22.0%) |  |
| 31,000 to 51,999 | 87008 (22.6%) | 22581 (23.4%) | 21865 (22.7%) | 21198 (22.0%) | 21364 (22.2%) |  |
| 52,000 to 100,000 | 68910 (17.9%) | 19919 (20.7%) | 17181 (17.8%) | 15878 (16.5%) | 15932 (16.5%) |  |
| Greater than 100,000 | 18413 (4.8%) | 6213 (6.5%) | 4727 (4.9%) | 3964 (4.1%) | 3509 (3.6%) |  |
| Missing | 55188 (14.3%) | 13843 (14.4%) | 13941 (14.5%) | 14082 (14.6%) | 13322 (13.8%) |  |
| Smoking status, n(%) |  |  |  |  |  | <0.001 |
| Never | 213314 (55.4%) | 58197 (60.4%) | 55459 (57.6%) | 52019 (54.0%) | 47639 (49.5%) |  |
| Previous | 131141 (34.0%) | 30811 (32.0%) | 31608 (32.8%) | 33697 (35.0%) | 35025 (36.4%) |  |
| Current | 40751 (10.6%) | 7294 (7.6%) | 9234 (9.6%) | 10585 (11.0%) | 13638 (14.2%) |  |
| Drinking status, n(%) |  |  |  |  |  | <0.001 |
| Never | 16877 (4.4%) | 3359 (3.5%) | 4146 (4.3%) | 4562 (4.7%) | 4810 (5.0%) |  |
| Previous | 13515 (3.5%) | 2429 (2.5%) | 3104 (3.2%) | 3689 (3.8%) | 4293 (4.5%) |  |
| Current | 354814 (92.1%) | 90514 (94.0%) | 89051 (92.5%) | 88050 (91.4%) | 87199 (90.5%) |  |
| Sleep Duration, hours/day | 7.15 ± 1.10 | 7.13 ± 1.02 | 7.14 ± 1.07 | 7.15 ± 1.12 | 7.16 ± 1.17 | <0.001 |
| IPAQ, n(%) |  |  |  |  |  | <0.001 |
| Low | 54942 (14.3%) | 10507 (10.9%) | 12552 (13.0%) | 14439 (15.0%) | 17444 (18.1%) |  |
| Moderate | 121166 (31.5%) | 30273 (31.4%) | 30313 (31.5%) | 30328 (31.5%) | 30252 (31.4%) |  |
| High | 122632 (31.8%) | 35141 (36.5%) | 31691 (32.9%) | 29289 (30.4%) | 26511 (27.5%) |  |
| Missing | 86466 (22.4%) | 20381 (21.2%) | 21745 (22.6%) | 22245 (23.1%) | 22095 (22.9%) |  |
| Meat intake (times/week), n(%) |  |  |  |  |  | <0.001 |
| Never | 35358 (9.2%) | 11933 (12.4%) | 9348 (9.7%) | 7807 (8.1%) | 6270 (6.5%) |  |
| Less than once a week | 117287 (30.4%) | 34218 (35.5%) | 31300 (32.5%) | 28012 (29.1%) | 23757 (24.7%) |  |
| Once a week | 111816 (29.0%) | 27105 (28.1%) | 27986 (29.1%) | 28614 (29.7%) | 28111 (29.2%) |  |
| 2-4 times a week | 105079 (27.3%) | 20288 (21.1%) | 24212 (25.1%) | 27840 (28.9%) | 32739 (34.0%) |  |
| 5-6 times a week | 12448 (3.2%) | 2193 (2.3%) | 2769 (2.9%) | 3211 (3.3%) | 4275 (4.4%) |  |
| Once or more daily | 3218 (0.8%) | 565 (0.6%) | 686 (0.7%) | 817 (0.8%) | 1150 (1.2%) |  |
| Fruit intake (pieces/day) | 2[1, 3] | 2[1, 3] | 2[1, 3] | 2[1, 3] | 2[1, 3] | <0.001 |
| Cereal intake (bowls/week) | 4.49 ± 2.8 | 4.4 ± 2.81 | 4.57 ± 2.78 | 4.56 ± 2.79 | 4.45 ± 2.81 | <0.001 |
| Vegetable intake (tablespoons/day) | 2 [2, 3] | 2 [2, 3] | 2 [2, 3] | 2 [2, 3] | 2 [2, 3] | <0.001 |
| Vitamin D intake, n (%) | 14771 (3.8%) | 4664 (4.8%) | 3853 (4.0%) | 3397 (3.5%) | 2857 (3.0%) | <0.001 |
| Diabetes, n(%) | 19881 (5.2%) | 2265 (2.4%) | 3354 (3.5%) | 5457 (5.7%) | 8805 (9.1%) | <0.001 |
| Hypertension, n(%) | 109749 (28.5%) | 18072 (18.8%) | 24833 (25.8%) | 30806 (32.0%) | 36038 (37.4%) | <0.001 |
| Family history of CRC, n(%) |  |  |  |  |  | <0.001 |
| No | 343793 (89.2%) | 86334 (89.6%) | 85998 (89.3%) | 85941 (89.2%) | 85520 (88.8%) |  |
| Yes | 41413 (10.8%) | 9968 (10.4%) | 10303 (10.7%) | 10360 (10.8%) | 10782 (11.2%) |  |
| BMI,kg/m^2^ | 27.42 ± 4.61 | 24.93 ± 3.77 | 26.82 ± 4.31 | 28.30 ± 4.50 | 29.63 ± 4.47 | <0.001 |
| NSAIDs, n(%) | 103854 (27.0%) | 22579 (23.4%) | 24647 (25.6%) | 27175 (28.2%) | 29453 (30.6%) | <0.001 |
| Lipid-lowering drug, n(%) | 66186 (17.2%) | 9535 (9.9%) | 13988 (14.5%) | 18869 (19.6%) | 23794 (24.7%) | <0.001 |
| Cancer screening, n(%) | 113352 (29.4%) | 25944 (26.9%) | 28682 (29.8%) | 29770 (30.9%) | 28956 (30.1%) | <0.001 |
| HbA1c,mmol/mol | 35.20  [32.90, 37.70] | 34.50  [32.20, 36.40] | 35.20  [32.70, 37.20] | 35.30  [33.20, 38.10] | 36.00  [33.70, 39.20] | <0.001 |
| Glucose, mmol/L | 4.93[4.60, 5.31] | 4.87 [4.56,5.21] | 4.91 [4.60,5.26] | 4.94 [4.62,5.34] | 4.98 [4.61,5.48] | <0.001 |
| TG,mmol/L | 1.48 [1.04,  2.15] | 0.84 [0.71, 0.99] | 1.25 [1.10, 1.43] | 1.75 [1.53, 2.00] | 2.79 [2.33, 3.48] | <0.001 |
| TC,mmol/L | 5.69 ± 1.11 | 5.61 ± 1.02 | 5.65 ± 1.08 | 5.71 ± 1.14 | 5.78 ± 1.20 | <0.001 |
| HDL-C,mmol/L | 1.44 ± 0.37 | 1.82 ±0.33 | 1.51 ± 0.27 | 1.32 ± 0.23 | 1.11 ± 0.20 | <0.001 |
| LDL-C,mmol/L | 3.56 ± 0.85 | 3.32 ± 0.76 | 3.54 ± 0.82 | 3.67 ± 0.87 | 3.71 ± 0.89 | <0.001 |
| CRP, mg/L | 1.32 [0.66, 2.68] | 0.86[0.44, 1.69] | 1.25[0.62, 2.45] | 1.51[0.81, 3.08] | 1.78 [0.98, 3.46] | <0.001 |
| IGF-1, nmol/L | 21.40 ± 5.41 | 21.51 ± 5.37 | 21.55 ± 5.37 | 21.42 ± 5.42 | 21.12 ± 5.46 | <0.001 |
| Fasting time, hours | 3[2, 4] | 3 [2, 5] | 3 [3, 4] | 3 [2, 4] | 3 [2, 4] | <0.001 |
| **Table S4** Baseline characteristics of the study population according to quartiles of METS-IR. | | | | | | |
| **Characteristic** | **Total** | **Quartiles of METS-IR** | | | | **P value** |
|  |  | **Q1**  **≤33.51** | **Q2**  **(33.51–38.98]** | **Q3**  **(38.98–45.54]** | **Q4**  **>45.54** |  |
| Number of participants | 385206 | 96302 | 96301 | 96301 | 96302 |  |
| Age, years | 56.24 ± 8.09 | 55.13 ± 8.13 | 56.46 ± 8.11 | 56.85 ± 8.06 | 56.51 ± 7.97 | <0.001 |
| Male, n(%) | 181710 (47.2%) | 25028 (26.0%) | 44354 (46.1%) | 55319 (57.4%) | 57009 (59.2%) | <0.001 |
| TDI | -1.31 ± 3.06 | -1.52 ± 2.96 | -1.53 ± 2.95 | -1.33 ± 3.05 | -0.86 ± 3.23 | <0.001 |
| Ethnicity, n(%) |  |  |  |  |  | <0.001 |
| White | 363548 (94.4%) | 91929 (95.5%) | 91073 (94.6%) | 90254 (93.7%) | 90292 (93.8%) |  |
| Others | 21658 (5.6%) | 4373 (4.5%) | 5228 (5.4%) | 6047 (6.3%) | 6010 (6.2%) |  |
| Qualification, n(%) |  |  |  |  |  | <0.001 |
| College | 125874 (32.7%) | 39618 (41.1%) | 33145 (34.4%) | 28992 (30.1%) | 24119 (25.0%) |  |
| Non-College | 259332 (67.3%) | 56684 (58.9%) | 63156 (65.6%) | 67309 (69.9%) | 72183 (75.0%) |  |
| Employment, n(%) |  |  |  |  |  | <0.001 |
| Yes | 228329 (59.3%) | 60203 (62.5%) | 57221 (59.4%) | 56154 (58.3%) | 54751 (56.9%) |  |
| No | 156877 (40.7%) | 36099 (37.5%) | 39080 (40.6%) | 40147 (41.7%) | 41551 (43.1%) |  |
| Income, n(%) |  |  |  |  |  | <0.001 |
| Less than 18,000 | 72918 (18.9%) | 15044 (15.6%) | 16678 (17.3%) | 18768 (19.5%) | 22428 (23.3%) |  |
| 18,000 to 30,999 | 82769 (21.5%) | 19387 (20.1%) | 20955 (21.8%) | 21204 (22.0%) | 21223 (22.0%) |  |
| 31,000 to 51,999 | 87008 (22.6%) | 22225 (23.1%) | 22287 (23.1%) | 21776 (22.6%) | 20720 (21.5%) |  |
| 52,000 to 100,000 | 68910 (17.9%) | 19643 (20.4%) | 17982 (18.7%) | 16889 (17.5%) | 14396 (14.9%) |  |
| Greater than 100,000 | 18413 (4.8%) | 6261 (6.5%) | 4896 (5.1%) | 4107 (4.3%) | 3149 (3.3%) |  |
| Missing | 55188 (14.3%) | 13742 (14.3%) | 13503 (14.0%) | 13557 (14.1%) | 14386 (14.9%) |  |
| Smoking status, n(%) |  |  |  |  |  | <0.001 |
| Never | 213314 (55.4%) | 58200 (60.4%) | 55073 (57.2%) | 51681 (53.7%) | 48360 (50.2%) |  |
| Previous | 131141 (34.0%) | 28337 (29.4%) | 31575 (32.8%) | 34243 (35.6%) | 36986 (38.4%) |  |
| Current | 40751 (10.6%) | 9765 (10.1%) | 9653 (10.0%) | 10377 (10.8%) | 10956 (11.4%) |  |
| Drinking status, n(%) |  |  |  |  |  | <0.001 |
| Never | 16877 (4.4%) | 3464 (3.6%) | 3664 (3.8%) | 4262 (4.4%) | 5487 (5.7%) |  |
| Previous | 13515 (3.5%) | 2667 (2.8%) | 2809 (2.9%) | 3248 (3.4%) | 4791 (5.0%) |  |
| Current | 354814 (92.1%) | 90171 (93.6%) | 89828 (93.3%) | 88791 (92.2%) | 86024 (89.3%) |  |
| Sleep Duration, hours/day | 7.15 ± 1.10 | 7.16 ± 1.00 | 7.16 ± 1.04 | 7.15 ± 1.10 | 7.12 ± 1.23 | <0.001 |
| IPAQ, n(%) |  |  |  |  |  | <0.001 |
| Low | 54942 (14.3%) | 10211 (10.6%) | 11780 (12.2%) | 14005 (14.5%) | 18946 (19.7%) |  |
| Moderate | 121166 (31.5%) | 31029 (32.2%) | 30755 (31.9%) | 30570 (31.7%) | 28812 (29.9%) |  |
| High | 122632 (31.8%) | 35523 (36.9%) | 33199 (34.5%) | 29868 (31.0%) | 24042 (25.0%) |  |
| Missing | 86466 (22.4%) | 19539 (20.3%) | 20567 (21.4%) | 21858 (22.7%) | 24502 (25.4%) |  |
| Meat intake (times/week), n(%) |  |  |  |  |  | <0.001 |
| Never | 35358 (9.2%) | 13736 (14.3%) | 8951 (9.3%) | 7022 (7.3%) | 5649 (5.9%) |  |
| Less than once a week | 117287 (30.4%) | 34090 (35.4%) | 30858 (32.0%) | 27682 (28.7%) | 24657 (25.6%) |  |
| Once a week | 111816 (29.0%) | 26188 (27.2%) | 28256 (29.3%) | 28850 (30.0%) | 28522 (29.6%) |  |
| 2-4 times a week | 105079 (27.3%) | 19464 (20.2%) | 24740 (25.7%) | 28537 (29.6%) | 32338 (33.6%) |  |
| 5-6 times a week | 12448 (3.2%) | 2278 (2.4%) | 2819 (2.9%) | 3327 (3.5%) | 4024 (4.2%) |  |
| Once or more daily | 3218 (0.8%) | 546 (0.6%) | 677 (0.7%) | 883 (0.9%) | 1112 (1.2%) |  |
| Fruit intake (pieces/day) | 2[1, 3] | 2[1, 3] | 2[1, 3] | 2[1, 3] | 2[1, 3] | <0.001 |
| Cereal intake (bowls/week) | 4.49 ± 2.80 | 4.58 ± 2.85 | 4.64 ± 2.79 | 4.48 ± 2.77 | 4.27 ± 2.77 | <0.001 |
| Vegetable intake (tablespoons/day) | 2 [2, 3] | 2 [2, 3] | 2 [2, 3] | 2 [2, 3] | 2 [2, 3] | <0.001 |
| Vitamin D intake, n (%) | 14771 (3.8%) | 5031 (5.2%) | 3801 (3.9%) | 3263 (3.4%) | 2676 (2.8%) | <0.001 |
| Diabetes, n(%) | 19881 (5.2%) | 1076 (1.1%) | 2097 (2.2%) | 4191 (4.4%) | 12517 (13.0%) | <0.001 |
| Hypertension, n(%) | 109749 (28.5%) | 13690 (14.2%) | 21911 (22.8%) | 30249 (31.4%) | 43899 (45.6%) | <0.001 |
| Family history of CRC, n(%) |  |  |  |  |  | <0.001 |
| No | 343793 (89.2%) | 86439 (89.8%) | 85930 (89.2%) | 85759 (89.1%) | 85665 (89.0%) |  |
| Yes | 41413 (10.8%) | 9863 (10.2%) | 10371 (10.8%) | 10542 (10.9%) | 10637 (11.0%) |  |
| BMI,kg/m^2^ | 27.42 ± 4.61 | 22.59 ± 1.76 | 25.68 ± 1.64 | 28.24 ± 2.00 | 33.17 ± 3.90 | <0.001 |
| NSAIDs, n(%) | 103854 (27.0%) | 20015 (20.8%) | 23343 (24.2%) | 26988 (28.0%) | 33508 (34.8%) | <0.001 |
| Lipid-lowering drug, n(%) | 66186 (17.2%) | 6763 (7.0%) | 12711 (13.2%) | 18923 (19.6%) | 27789 (28.9%) | <0.001 |
| Cancer screening, n(%) | 113352 (29.4%) | 26164 (27.2%) | 28808 (29.9%) | 29501 (30.6%) | 28879 (30.0%) | <0.001 |
| HbA1c,mmol/mol | 35.20  [32.90, 37.70] | 34.40  [32.20, 36.30] | 35.00  [32.60, 37.00] | 35.20  [33.10, 37.80] | 36.50  [34.20, 40.20] | <0.001 |
| Glucose, mmol/L | 4.93[4.60, 5.31] | 4.82[4.50, 5.14] | 4.90[4.59, 5.24] | 4.95[4.63, 5.32] | 5.07[4.69, 5.63] | <0.001 |
| TG,mmol/L | 1.48[1.04, 2.14] | 0.99[0.78, 1.28] | 1.34[1.02, 1.77] | 1.73[1.28, 2.33] | 2.24[1.62, 3.11] | <0.001 |
| TC,mmol/L | 5.69 ± 1.11 | 5.74 ± 1.03 | 5.76 ± 1.09 | 5.73 ± 1.14 | 5.51± 1.18 | <0.001 |
| HDL-C,mmol/L | 1.44 ± 0.37 | 1.79 ±0.34 | 1.51 ± 0.29 | 1.33 ± 0.26 | 1.16 ± 0.24 | <0.001 |
| LDL-C,mmol/L | 3.56 ± 0.85 | 3.43 ± 0.78 | 3.62 ± 0.83 | 3.66 ± 0.87 | 3.52 ± 0.89 | <0.001 |
| CRP, mg/L | 1.32 [0.66, 2.68] | 0.71 [0.38, 1.34] | 1.13 [0.61, 2.08] | 1.50 [0.85, 2.84] | 2.37 [1.32, 4.56] | <0.001 |
| IGF-1, nmol/L | 21.40 ± 5.41 | 21.59 ± 5.32 | 21.94 ± 5.28 | 21.74 ± 5.35 | 20.33 ± 5.53 | <0.001 |
| Fasting time, hours | 3[2, 4] | 3[2, 4] | 3[2, 4] | 3[2, 4] | 3[2, 4] | <0.001 |

| **Table S5** Associations between the four IR surrogate markers and incident CRC risk, stratified by anatomical sub-site (colon cancer and rectal cancer), based on the fully adjusted Model 4. | | | | |
| --- | --- | --- | --- | --- |
| **Type** | **colon cancer** | | **rectal cancer** | |
|  | **HR (95% CI)** | **P value** | **HR (95% CI)** | **P value** |
| **TyG** |  |  |  |  |
| Q1 | Ref | Ref | Ref | Ref |
| Q2 | 0.96 (0.86-1.06) | 0.389 | 1.00 (0.88-1.15) | 0.966 |
| Q3 | 1.03 (0.94-1.14) | 0.5 | 0.99 (0.87-1.13) | 0.904 |
| Q4 | 1.19 (1.08-1.31) | <0.001 | 1.09 (0.96-1.25) | 0.184 |
| P for trend | <0.001 |  | 0.184 |  |
| Per SD increase | 1.08 (1.04-1.12) | <0.001 | 1.05 (1.00-1.10) | 0.051 |
| **TyG-BMI** |  |  |  |  |
| Q1 | Ref | Ref | Ref | Ref |
| Q2 | 1.01 (0.91-1.12) | 0.862 | 0.96 (0.84-1.10) | 0.572 |
| Q3 | 1.10 (0.99-1.21) | 0.076 | 0.98 (0.86-1.12) | 0.795 |
| Q4 | 1.25 (1.12-1.38) | <0.001 | 1.13 (0.98-1.29) | 0.092 |
| P for trend | <0.001 |  | 0.059 |  |
| Per SD increase | 1.11 (1.07-1.15) | <0.001 | 1.06 (1.01-1.11) | 0.017 |
| **TG/HDL** |  |  |  |  |
| Q1 | Ref | Ref | Ref | Ref |
| Q2 | 0.93 (0.84-1.03) | 0.178 | 0.94 (0.82-1.08) | 0.383 |
| Q3 | 1.04 (0.94-1.14) | 0.484 | 0.98 (0.86-1.12) | 0.803 |
| Q4 | 1.14 (1.03-1.26) | 0.01 | 1.02 (0.89-1.16) | 0.818 |
| P for trend | <0.001 |  | 0.598 |  |
| Per SD increase | 1.08 (1.05-1.12) | <0.001 | 1.02 (0.98-1.07) | 0.322 |
| **METS-IR** |  |  |  |  |
| Q1 | Ref | Ref | Ref | Ref |
| Q2 | 1.03 (0.93-1.15) | 0.519 | 0.94 (0.82-1.08) | 0.379 |
| Q3 | 1.08 (0.97-1.19) | 0.16 | 0.93 (0.81-1.07) | 0.319 |
| Q4 | 1.29 (1.16-1.43) | <0.001 | 1.10 (0.96-1.27) | 0.17 |
| P for trend | <0.001 |  | 0.129 |  |
| Per SD increase | 1.12 (1.08-1.16) | <0.001 | 1.05 (1.00-1.11) | 0.038 |

| **Table S6** Sensitivity analysis for the associations between the four IR surrogate markers and incident CRC risk, excluding participants who developed CRC within the first two years of follow-up. | | | | |
| --- | --- | --- | --- | --- |
| **Type** | **Model 1** | **Model 2** | **Model 3** | **Model 4** |
|  | **HR (95% CI)** | **HR (95% CI)** | **HR (95% CI)** | **HR (95% CI)** |
| **TyG** |  |  |  |  |
| Q1 | Ref | Ref | Ref | Ref |
| Q2 | 1.16 (1.06-1.26)^b^ | 0.96 (0.88-1.05) | 0.96 (0.87-1.04) | 0.96 (0.88-1.05) |
| Q3 | 1.32 (1.21-1.44)^c^ | 1.03 (0.94-1.12) | 1.01 (0.92-1.10) | 1.01 (0.93-1.11) |
| Q4 | 1.57 (1.45-1.71)^c^ | 1.19 (1.09-1.30)^c^ | 1.16 (1.06-1.26)^c^ | 1.16 (1.07-1.27)^c^ |
| P for trend | <0.001 | <0.001 | <0.001 | <0.001 |
| Per SD increase | 1.19 (1.16-1.23)^c^ | 1.09 (1.06-1.12)^c^ | 1.08 (1.04-1.11)^c^ | 1.08 (1.04-1.11)^c^ |
| KM Cutpoint |  |  |  |  |
| ≤9.12 | Ref | Ref | Ref | Ref |
| ＞9.12 | 1.40 (1.31-1.49)^c^ | 1.24 (1.16-1.32)^c^ | 1.21 (1.13-1.29)^c^ | 1.21 (1.14-1.30)^c^ |
| **TyG-BMI** |  |  |  |  |
| Q1 | Ref | Ref | Ref | Ref |
| Q2 | 1.23 (1.13-1.35)^c^ | 1.03 (0.94-1.12) | 1.02 (0.93-1.11) | 1.02 (0.93-1.11) |
| Q3 | 1.39 (1.27-1.51)^c^ | 1.10 (1.00-1.20)^a^ | 1.07 (0.98-1.17) | 1.07 (0.98-1.17) |
| Q4 | 1.61 (1.47-1.75)^c^ | 1.32 (1.21-1.43)^c^ | 1.26 (1.16-1.38)^c^ | 1.26 (1.15-1.38)^c^ |
| P for trend | <0.001 | <0.001 | <0.001 | <0.001 |
| Per SD increase | 1.17 (1.14-1.21)^c^ | 1.12 (1.09-1.16)^c^ | 1.11 (1.07-1.14)^c^ | 1.11 (1.07-1.14)^c^ |
| KM Cutpoint |  |  |  |  |
| ≤218.52 | Ref | Ref | Ref | Ref |
| ＞218.52 | 1.41 (1.32-1.50)^c^ | 1.20 (1.12-1.28)^c^ | 1.16 (1.09-1.24)^c^ | 1.16 (1.08-1.24)^c^ |
| **TG/HDL** |  |  |  |  |
| Q1 | Ref | Ref | Ref | Ref |
| Q2 | 1.04 (0.95-1.14) | 0.91 (0.83-0.99)^a^ | 0.90 (0.83-0.99)^a^ | 0.91 (0.83-0.99)^a^ |
| Q3 | 1.24 (1.14-1.35)^c^ | 1.00 (0.91-1.09) | 0.99 (0.90-1.08) | 0.99 (0.91-1.08) |
| Q4 | 1.42 (1.31-1.54)^c^ | 1.13 (1.04-1.23)^b^ | 1.10 (1.01-1.20)^a^ | 1.11 (1.01-1.21)^a^ |
| P for trend | <0.001 | <0.001 | 0.002 | 0.002 |
| Per SD increase | 1.14 (1.11-1.17)^c^ | 1.09 (1.06-1.12)^c^ | 1.08 (1.05-1.11)^c^ | 1.08 (1.05-1.11)^c^ |
| KM Cutpoint |  |  |  |  |
| ≤2.61 | Ref | Ref | Ref | Ref |
| ＞2.61 | 1.33 (1.26-1.41)^c^ | 1.16 (1.09-1.23)^c^ | 1.14 (1.07-1.21)^c^ | 1.14 (1.07-1.21)^c^ |
| **METS-IR** |  |  |  |  |
| Q1 | Ref | Ref | Ref | Ref |
| Q2 | 1.18 (1.08-1.28)^c^ | 1.01 (0.92-1.10) | 1.00 (0.91-1.10) | 1.00 (0.92-1.10) |
| Q3 | 1.29 (1.18-1.41)^c^ | 1.04 (0.95-1.14) | 1.02 (0.94-1.12) | 1.02 (0.94-1.12) |
| Q4 | 1.56 (1.44-1.70)^c^ | 1.30 (1.19-1.42)^c^ | 1.25 (1.15-1.37)^c^ | 1.26 (1.15-1.38)^c^ |
| P for trend | <0.001 | <0.001 | <0.001 | <0.001 |
| Per SD increase | 1.17 (1.14-1.20)^c^ | 1.12 (1.09-1.16)^c^ | 1.11 (1.08-1.15)^c^ | 1.11 (1.08-1.15)^c^ |
| KM Cutpoint |  |  |  |  |
| ≤44.79 | Ref | Ref | Ref | Ref |
| ＞44.79 | 1.38 (1.30-1.47)^c^ | 1.29 (1.21-1.38)^c^ | 1.26 (1.18-1.35)^c^ | 1.26 (1.18-1.35)^c^ |
| **Table S7** Sensitivity analysis for the associations between the four IR surrogate markers and incident CRC risk, excluding participants who died from any cause within the first two years of follow-up. | | | | |
| **Type** | **Model 1** | **Model 2** | **Model 3** | **Model 4** |
|  | **HR (95% CI)** | **HR (95% CI)** | **HR (95% CI)** | **HR (95% CI)** |
| **TyG** |  |  |  |  |
| Q1 | Ref | Ref | Ref | Ref |
| Q2 | 1.19 (1.10-1.30)^c^ | 0.99 (0.91-1.08) | 0.98 (0.90-1.07) | 0.99 (0.91-1.07) |
| Q3 | 1.35 (1.25-1.47)^c^ | 1.05 (0.96-1.14) | 1.03 (0.95-1.12) | 1.03 (0.95-1.12) |
| Q4 | 1.59 (1.47-1.72)^c^ | 1.20 (1.10-1.30)^c^ | 1.16 (1.07-1.26)^c^ | 1.17 (1.08-1.27)^c^ |
| P for trend | <0.001 | <0.001 | <0.001 | <0.001 |
| Per SD increase | 1.19 (1.16-1.22)^c^ | 1.08 (1.05-1.11)^c^ | 1.07 (1.04-1.10)^c^ | 1.07 (1.04-1.10)^c^ |
| KM Cutpoint |  |  |  |  |
| ≤8.68 | Ref | Ref | Ref | Ref |
| ＞8.68 | 1.34 (1.27-1.42)^c^ | 1.13 (1.07-1.19)^c^ | 1.11 (1.05-1.17)^c^ | 1.11 (1.05-1.17)^c^ |
| **TyG-BMI** |  |  |  |  |
| Q1 | Ref | Ref | Ref | Ref |
| Q2 | 1.23 (1.13-1.33)^c^ | 1.02 (0.94-1.11) | 1.01 (0.93-1.10) | 1.01 (0.92-1.10) |
| Q3 | 1.41 (1.30-1.52)^c^ | 1.11 (1.02-1.20)^a^ | 1.08 (0.99-1.17) | 1.08 (0.99-1.17) |
| Q4 | 1.56 (1.44-1.69)^c^ | 1.27 (1.17-1.38)^c^ | 1.22 (1.12-1.33)^c^ | 1.22 (1.12-1.32)^c^ |
| P for trend | <0.001 | <0.001 | <0.001 | <0.001 |
| Per SD increase | 1.16 (1.13-1.19)^c^ | 1.11 (1.08-1.14)^c^ | 1.09 (1.06-1.13)^c^ | 1.09 (1.06-1.13)^c^ |
| KM Cutpoint |  |  |  |  |
| ≤218.40 | Ref | Ref | Ref | Ref |
| ＞218.40 | 1.41 (1.32-1.49)^c^ | 1.19 (1.12-1.27)^c^ | 1.16 (1.09-1.23)^c^ | 1.15 (1.08-1.23)^c^ |
| **TG/HDL** |  |  |  |  |
| Q1 | Ref | Ref | Ref | Ref |
| Q2 | 1.07 (0.99-1.16) | 0.93 (0.86-1.01) | 0.93 (0.85-1.01) | 0.93 (0.85-1.01) |
| Q3 | 1.28 (1.18-1.38)^c^ | 1.03 (0.95-1.11) | 1.02 (0.94-1.10) | 1.02 (0.94-1.10) |
| Q4 | 1.42 (1.31-1.54)^c^ | 1.12 (1.04-1.22)^b^ | 1.10 (1.01-1.19)^a^ | 1.10 (1.01-1.20)^a^ |
| P for trend | <0.001 | <0.001 | 0.002 | 0.002 |
| Per SD increase | 1.13 (1.10-1.16)^c^ | 1.08 (1.05-1.10)^c^ | 1.07 (1.04-1.10)^c^ | 1.07 (1.04-1.10)^c^ |
| KM Cutpoint |  |  |  |  |
| ≤2.61 | Ref | Ref | Ref | Ref |
| ＞2.61 | 1.33 (1.26-1.40)^c^ | 1.14 (1.08-1.21)^c^ | 1.13 (1.07-1.19)^c^ | 1.13 (1.07-1.20)^c^ |
| **METS-IR** |  |  |  |  |
| Q1 | Ref | Ref | Ref | Ref |
| Q2 | 1.19 (1.09-1.29)^c^ | 1.01 (0.93-1.10) | 1.01 (0.93-1.10) | 1.01 (0.93-1.10) |
| Q3 | 1.31 (1.21-1.42)^c^ | 1.05 (0.97-1.15) | 1.03 (0.95-1.12) | 1.03 (0.95-1.12) |
| Q4 | 1.54 (1.42-1.66)^c^ | 1.27 (1.17-1.37)^c^ | 1.22 (1.13-1.33)^c^ | 1.22 (1.12-1.33)^c^ |
| P for trend | <0.001 | <0.001 | <0.001 | <0.001 |
| Per SD increase | 1.16 (1.13-1.19)^c^ | 1.11 (1.08-1.14)^c^ | 1.10 (1.07-1.13)^c^ | 1.10 (1.06-1.13)^c^ |
| KM Cutpoint |  |  |  |  |
| ≤44.79 | Ref | Ref | Ref | Ref |
| ＞44.79 | 1.35 (1.27-1.43)^c^ | 1.26 (1.19-1.33)^c^ | 1.23 (1.16-1.31)^c^ | 1.23 (1.15-1.31)^c^ |
| **Table S8** Sensitivity analysis for the associations between the four IR surrogate markers and incident CRC risk among participants with complete baseline covariate data (complete-case analysis). | | | | |
| **Type** | **Model 1** | **Model 2** | **Model 3** | **Model 4** |
|  | **HR (95% CI)** | **HR (95% CI)** | **HR (95% CI)** | **HR (95% CI)** |
| **TyG** |  |  |  |  |
| Q1 | Ref | Ref | Ref | Ref |
| Q2 | 1.26 (1.14-1.40)^c^ | 1.04 (0.94-1.16) | 1.02 (0.91-1.13) | 1.02 (0.91-1.13) |
| Q3 | 1.43 (1.29-1.58)^c^ | 1.08 (0.98-1.20) | 1.06 (0.95-1.18) | 1.07 (0.96-1.19) |
| Q4 | 1.67 (1.51-1.85)^c^ | 1.24 (1.12-1.37)^c^ | 1.20 (1.08-1.34)^c^ | 1.21 (1.09-1.34)^c^ |
| P for trend | <0.001 | <0.001 | <0.001 | 0.002 |
| Per SD increase | 1.22 (1.18-1.26)^c^ | 1.11 (1.07-1.15)^c^ | 1.10 (1.06-1.14)^c^ | 1.10 (1.06-1.14)^c^ |
| KM Cutpoint |  |  |  |  |
| ≤8.62 | Ref | Ref | Ref | Ref |
| ＞8.62 | 1.42 (1.32-1.52)^c^ | 1.17 (1.09-1.25)^c^ | 1.15 (1.07-1.24)^c^ | 1.15 (1.07-1.24)^c^ |
| **TyG-BMI** |  |  |  |  |
| Q1 | Ref | Ref | Ref | Ref |
| Q2 | 1.26 (1.14-1.40)^c^ | 1.04 (0.93-1.15) | 1.05 (0.94-1.17) | 1.05 (0.94-1.17) |
| Q3 | 1.48 (1.33-1.63)^c^ | 1.14 (1.03-1.27)^a^ | 1.13 (1.02-1.26)^a^ | 1.13 (1.01-1.26)^a^ |
| Q4 | 1.63 (1.47-1.80)^c^ | 1.31 (1.19-1.46)^c^ | 1.27 (1.14-1.41)^c^ | 1.26 (1.13-1.40)^c^ |
| P for trend | <0.001 | <0.001 | <0.001 | <0.001 |
| Per SD increase | 1.18 (1.14-1.22)^c^ | 1.13 (1.09-1.17)^c^ | 1.11 (1.07-1.15)^c^ | 1.11 (1.07-1.15)^c^ |
| KM Cutpoint |  |  |  |  |
| ≤218.44 | Ref | Ref | Ref | Ref |
| ＞218.44 | 1.46 (1.36-1.57)^c^ | 1.23 (1.14-1.33)^c^ | 1.21 (1.12-1.31)^c^ | 1.20 (1.11-1.30)^c^ |
| **TG/HDL** |  |  |  |  |
| Q1 | Ref | Ref | Ref | Ref |
| Q2 | 1.10 (0.99-1.22) | 0.94 (0.85-1.04) | 0.92 (0.83-1.03) | 0.92 (0.83-1.03) |
| Q3 | 1.34 (1.21-1.48)^c^ | 1.05 (0.95-1.17) | 1.04 (0.94-1.15) | 1.04 (0.94-1.15) |
| Q4 | 1.48 (1.34-1.63)^c^ | 1.15 (1.03-1.27)^b^ | 1.11 (1.00-1.24)^a^ | 1.11 (1.00-1.24)^a^ |
| P for trend | <0.001 | <0.001 | 0.005 | 0.005 |
| Per SD increase | 1.14 (1.11-1.18)^c^ | 1.09 (1.05-1.12)^c^ | 1.08 (1.04-1.11)^c^ | 1.07 (1.04-1.11)^c^ |
| KM Cutpoint |  |  |  |  |
| ≤2.61 | Ref | Ref | Ref | Ref |
| ＞2.61 | 1.36 (1.27-1.46)^c^ | 1.16 (1.09-1.25)^c^ | 1.15 (1.07-1.24)^c^ | 1.15 (1.07-1.24)^c^ |
| **METS-IR** |  |  |  |  |
| Q1 | Ref | Ref | Ref | Ref |
| Q2 | 1.22 (1.10-1.35)^c^ | 1.03 (0.93-1.14) | 1.04 (0.94-1.16) | 1.04 (0.94-1.16) |
| Q3 | 1.30 (1.17-1.44)^c^ | 1.03 (0.93-1.14) | 1.03 (0.92-1.15) | 1.02 (0.92-1.14) |
| Q4 | 1.63 (1.48-1.80)^c^ | 1.32 (1.20-1.47)^c^ | 1.29 (1.16-1.44)^c^ | 1.28 (1.15-1.43)^c^ |
| P for trend | <0.001 | <0.001 | <0.001 | <0.001 |
| Per SD increase | 1.18 (1.14-1.21)^c^ | 1.12 (1.08-1.16)^c^ | 1.11 (1.07-1.15)^c^ | 1.10 (1.06-1.14)^c^ |
| KM Cutpoint |  |  |  |  |
| ≤44.79 | Ref | Ref | Ref | Ref |
| ＞44.79 | 1.40 (1.30-1.50)^c^ | 1.30 (1.21-1.40)^c^ | 1.26 (1.17-1.36)^c^ | 1.25 (1.16-1.36)^c^ |
| **Table S9** Sensitivity analysis for the associations between the four IR surrogate markers and incident CRC risk after addressing missing covariates via multiple imputation by chained equations (MICE). | | | | |
| **Type** | **Model 1** | **Model 2** | **Model 3** | **Model 4** |
|  | **HR (95% CI)** | **HR (95% CI)** | **HR (95% CI)** | **HR (95% CI)** |
| **TyG** |  |  |  |  |
| Q1 | Ref | Ref | Ref | Ref |
| Q2 | 1.19 (1.09-1.29)^c^ | 0.97 (0.89-1.07) | 1.01 (0.92-1.12) | 1.02 (0.92-1.13) |
| Q3 | 1.35 (1.25-1.47)^c^ | 1.05 (0.96-1.15) | 1.07 (0.97-1.18) | 1.07 (0.97-1.18) |
| Q4 | 1.58 (1.46-1.71)^c^ | 1.17 (1.07-1.28)^c^ | 1.19 (1.08-1.31)^c^ | 1.21 (1.09-1.33)^c^ |
| P for trend | <0.001 | <0.001 | <0.001 | 0.001 |
| Per SD increase | 1.19 (1.16-1.22)^c^ | 1.08 (1.05-1.12)^c^ | 1.09 (1.05-1.12)^c^ | 1.09 (1.05-1.13)^c^ |
| KM Cutpoint |  |  |  |  |
| ≤8.68 | Ref | Ref | Ref | Ref |
| ＞8.68 | 1.34 (1.27-1.42)^c^ | 1.13 (1.06-1.20)^c^ | 1.12 (1.05-1.20)^b^ | 1.13 (1.05-1.21)^c^ |
| **TyG-BMI** |  |  |  |  |
| Q1 | Ref | Ref | Ref | Ref |
| Q2 | 1.22 (1.12-1.32)^c^ | 1.03 (0.94-1.13) | 1.00 (0.91-1.11) | 0.99 (0.89-1.10) |
| Q3 | 1.40 (1.29-1.51)^c^ | 1.14 (1.04-1.25)^b^ | 1.11 (1.01-1.23)^a^ | 1.10 (1.00-1.22) |
| Q4 | 1.55 (1.43-1.68)^c^ | 1.27 (1.16-1.39)^c^ | 1.25 (1.13-1.38)^c^ | 1.23 (1.11-1.37)^c^ |
| P for trend | <0.001 | <0.001 | <0.001 | <0.001 |
| Per SD increase | 1.16 (1.13-1.19)^c^ | 1.11 (1.08-1.15)^c^ | 1.11 (1.07-1.15)^c^ | 1.11 (1.07-1.15)^c^ |
| KM Cutpoint |  |  |  |  |
| ≤218.40 | Ref | Ref | Ref | Ref |
| ＞218.40 | 1.40 (1.32-1.49)^c^ | 1.20 (1.12-1.28)^c^ | 1.20 (1.11-1.29)^c^ | 1.18 (1.10-1.28)^c^ |
| **TG/HDL** |  |  |  |  |
| Q1 | Ref | Ref | Ref | Ref |
| Q2 | 1.07 (0.99-1.16) | 0.91 (0.83-1.00) | 0.92 (0.83-1.01) | 0.92 (0.83-1.02) |
| Q3 | 1.28 (1.18-1.38)^c^ | 1.02 (0.93-1.11) | 1.02 (0.92-1.13) | 1.03 (0.93-1.14) |
| Q4 | 1.42 (1.31-1.53)^c^ | 1.10 (1.00-1.20)^a^ | 1.09 (0.99-1.21) | 1.11 (1.00-1.23) |
| P for trend | <0.001 | 0.003 | 0.012 | 0.006 |
| Per SD increase | 1.13 (1.10-1.16)^c^ | 1.07 (1.04-1.10)^c^ | 1.07 (1.03-1.10)^c^ | 1.07 (1.04-1.11)^c^ |
| KM Cutpoint |  |  |  |  |
| ≤2.61 | Ref | Ref | Ref | Ref |
| ＞2.61 | 1.32 (1.25-1.40)^c^ | 1.13 (1.07-1.21)^c^ | 1.13 (1.05-1.21)^c^ | 1.14 (1.06-1.22)^c^ |
| **METS-IR** |  |  |  |  |
| Q1 | Ref | Ref | Ref | Ref |
| Q2 | 1.18 (1.09-1.29)^c^ | 1.03 (0.94-1.13) | 1.03 (0.93-1.14) | 1.02 (0.92-1.13) |
| Q3 | 1.31 (1.20-1.42)^c^ | 1.06 (0.97-1.16) | 1.06 (0.95-1.17) | 1.04 (0.94-1.16) |
| Q4 | 1.53 (1.42-1.66)^c^ | 1.27 (1.16-1.39)^c^ | 1.26 (1.14-1.40)^c^ | 1.25 (1.12-1.39)^c^ |
| P for trend | <0.001 | <0.001 | <0.001 | <0.001 |
| Per SD increase | 1.16 (1.13-1.19)^c^ | 1.11 (1.07-1.14)^c^ | 1.10 (1.06-1.14)^c^ | 1.10 (1.06-1.14)^c^ |
| KM Cutpoint |  |  |  |  |
| ≤44.79 | Ref | Ref | Ref | Ref |
| ＞44.79 | 1.35 (1.28-1.43)^c^ | 1.25 (1.17-1.33)^c^ | 1.25 (1.16-1.34)^c^ | 1.25 (1.16-1.35)^c^ |

| **Table S10** Sensitivity analysis for the associations between the four IR surrogate markers and incident CRC risk after additional adjustment for potential clinical and biochemical mediators (Model 5). | | | |
| --- | --- | --- | --- |
| **Type** | **HR (95% CI)** | **P value** | |
| **TyG** | | |  |
| Q1 | Ref |  | |
| Q2 | 0.99 (0.91-1.07) | 0.741 | |
| Q3 | 1.04 (0.95-1.13) | 0.37 | |
| Q4 | 1.16 (1.07-1.27) | <0.001 | |
| P for trend | <0.001 |  | |
| Per SD increase | 1.07 (1.04-1.10) | <0.001 | |
| KM Cutpoint |  |  |  |
| ≤8.68 | Ref |  | |
| ＞8.68 | 1.11 (1.04-1.17) | 0.001 | |
| **TyG-BMI** | | | |
| Q1 | Ref |  | |
| Q2 | 1.00 (0.92-1.09) | 0.949 | |
| Q3 | 1.07 (0.98-1.17) | 0.128 | |
| Q4 | 1.19 (1.08-1.30) | <0.001 | |
| P for trend | <0.001 |  | |
| Per SD increase | 1.08 (1.05-1.12) | <0.001 | |
| KM Cutpoint |  |  | |
| ≤218.40 | Ref |  | |
| ＞218.40 | 1.14 (1.07-1.22) | <0.001 | |
| **TG/HDL** |  |  | |
| Q1 | Ref |  | |
| Q2 | 0.94 (0.86-1.02) | 0.153 | |
| Q3 | 1.03 (0.94-1.12) | 0.569 | |
| Q4 | 1.10 (1.01-1.20) | 0.037 | |
| P for trend | 0.003 |  | |
| Per SD increase | 1.06 (1.03-1.09) | <0.001 | |
| KM Cutpoint |  |  | |
| ≤2.61 | Ref |  | |
| ＞2.61 | 1.13 (1.07-1.20) | <0.001 | |
| **METS-IR** | | | |
| Q1 | Ref |  | |
| Q2 | 1.01 (0.93-1.11) | 0.741 | |
| Q3 | 1.04 (0.95-1.14) | 0.363 | |
| Q4 | 1.22 (1.11-1.34) | <0.001 | |
| P for trend | <0.001 |  | |
| Per SD increase | 1.10 (1.06-1.13) | <0.001 | |
| KM Cutpoint |  |  | |
| ≤44.79 | Ref |  | |
| ＞44.79 | 1.22 (1.14-1.30) | <0.001 | |


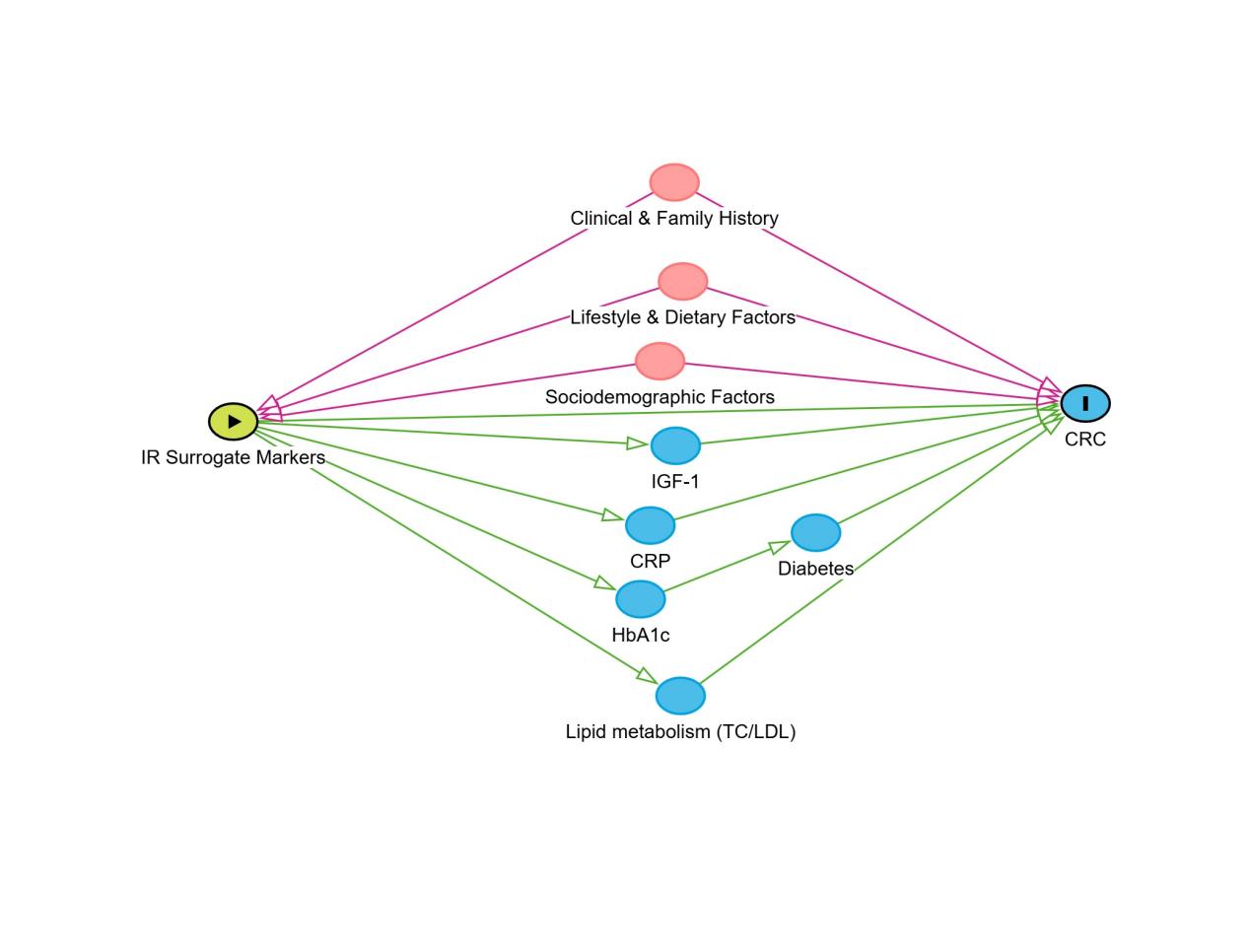


Figure S1. Directed Acyclic Graph (DAG) of the association between insulin resistance (IR) surrogate markers and colorectal cancer (CRC) risk.

To maintain visual clarity, potential confounders were grouped into three thematic modules: Sociodemographic Factors (Model 2: age, sex, annual household income, ethnicity, education level, employment status, and TDI), Lifestyle & Dietary Factors (Model 3: physical activity level, sleep duration, smoking status, drinking status, and dietary intakes of meat, fruit, cereal, and vegetables and vitamin D supplementation), and Clinical & Family History (Model 4 components: hypertension, family history of CRC, fasting duration, the regular use of lipid-lowering medications and NSAIDs, and bowel cancer screening history). These factors are identified as ancestral confounders that influence both metabolic status and CRC risk. Clinical and biochemical indicators (IGF-1, CRP, HbA1c, Diabetes, and Lipid metabolism) are hypothesized as downstream mediators or manifestations. The DAG justifies our stepwise modeling strategy, distinguishing between the total metabolic effect (adjusting for confounders only) and the independent direct effect (further adjusting for mediating pathways).
